# Learning about COVID-19-related stigma, quarantine and isolation experiences in Finland

**DOI:** 10.1371/journal.pone.0247962

**Published:** 2021-04-14

**Authors:** Anna-Leena Lohiniva, Timothee Dub, Lotta Hagberg, Hanna Nohynek

**Affiliations:** The Finnish Institute for Health and Welfare, Helsinki, Finland; University of North Carolina at Greensboro, UNITED STATES

## Abstract

**Background:**

The COVID-19 pandemic has intensely changed the everyday lives of people worldwide. This study explores the forms and outcomes of coronavirus and COVID-19-related social stigma and the experiences of people who were home quarantined or isolated in Finland during the spring 2020. The findings of this study can be used to improve support for those quarantined or isolated and to develop strategies to reduce the stigma associated with coronavirus and COVID-19.

**Methods:**

The study is based on qualitative one-to-one interviews with households with at least two members and at least one PCR confirmed COVID-19 case. Recruitment took place via website or SMS messages sent to PCR confirmed cases in the capital area of Helsinki. Sampling was based on maximum variation to acquire different types of respondents. The framework of health stigma was used to develop question guides and analyze stigma. Quarantine and isolation experiences were explored through open-ended questions. The analysis was based on thematic analysis.

**Results:**

The study included 64 participants from 24 households. Perceived stigma among respondents was driven by fear and blame for infection, and it manifested in various ways leading to a reluctance to disclose their coronavirus status to others. Self-stigma developed from conflicting information and advice about coronavirus and COVID-19 led to difficulties interacting with others outside of the house and reluctance to meet people after quarantine and isolation. Quarantine and isolation experiences included uncertainty, health concerns, and boredom. Communication with others in similar situations was perceived vital, whereas discussions with family members about worries and fears related to coronavirus and COVID-19 was not preferred.

**Conclusions:**

This study shed light on the lives of those quarantined or isolated at home and provided a set of operational recommendations to minimize coronavirus and COVID-19-associated stigma and to reduce challenges faced by those in quarantine or isolation.

## Introduction

The coronavirus SARS- CoV-2 that causes the disease COVID‐19 has caused significant disruption in everyday life on a global scale. Several public health measures have been put in place to reduce the transmission of the virus and to minimize the impact of the disease [1, 2]. These measures include isolation of those who have acquired the infection and quarantine of their close contacts [3]. Quarantine refers to separating and restricting the movement of people who are exposed to a contagious disease to see if they become sick whereas isolation means separating infected people from those who are not known to be infected [4].

The effect of various public health measures to contain the COVID-19 pandemic is not yet fully understood [3]. However, recent studies increasingly support quarantine measures as an effective precaution to curb transmission of the virus [5–7]. Moreover, quarantine appeared to be an effective virus mitigation measure in several countries during previous SARS outbreaks in 2003 including Singapore, Canada and China [8–10]. There are various kinds of isolation and quarantine measures as well as systems to follow up on the implementation of these measures worldwide [11]. In Finland, isolation and quarantine measures are home based with an official notification about the measures and regular phone-based follow up by health officials. Quarantined persons without symptoms are usually not tested. Persons in quarantine are allowed to go out in public as long as they are not in contact with other people. However, going to work, school, or shopping is not permitted during quarantine [12]. This is based on the Communicable Diseases Act Infectious Disease Law that stipulates that both quarantine and isolation are obligatory [13]. Breach of the law is punishable by up to three months of imprisonment. However, follow up on the adherence of the law is not systematic.

Those in quarantine found themselves isolated at home without their usual routines. This rise in unstructured time, combined with the enormous stress of the pandemic and its far‐reaching consequences on both health and the economy, has led to widespread concerns including worry about social stigma [14]. Stigma is a well-documented global barrier to health-seeking behavior, engagement in care, and adherence to treatment across a range of health conditions including infectious diseases [15–18]. It may also occur due to patient isolation and quarantine procedures [19, 20].

Stigma operates across different levels including in the public sphere through public policies, in organizations, and in communities through cultural values, norm, and attitudes in communities. Stigma also operates in interpersonal relations related to family and friends, and on an individual level related to the knowledge and skills of individuals to manage stigma [21–24]. Although previous studies indicate many similarities regarding stigma across countries, stigma is also known to be context-specific which necessitates understanding stigma in the given context [25]. Coronavirus and COVID-19-related stigma has been reported in number of countries such as in India, Ethiopia, Japan and Uganda [26–31]. Stigma against healthcare workers associated with the care of coronavirus patients has been reported widely as well [32]. However, little is known about the nature of coronavirus and COVID-19-related stigma, which is paramount for developing effective stigma reduction strategies.

Several studies have investigated the psychological experience of quarantined individuals during major infectious disease outbreaks [33–35], but less focus has been given to social and behavioral research that is necessary to develop meaningful support strategies for those isolated or quarantined [36]. This study aims to review the forms, drivers, outcomes, and impact of social stigma towards those with coronavirus and COVID-19 and their family members, and to shed light on their quarantine experiences in order to develop operational recommendations for public health officials in Finland and other similar cultural settings to better support people during and after quarantine or isolation.

## Materials and methods

This study is comprised of in-depth interviews with people from households that experienced coronavirus and were in home quarantine or in isolation for a period of time. The interviews were conducted between April and May 2020 by a researcher (A-LL) trained and experienced in qualitative data collection and analysis methods. Those eligible to join the study were people from households located in the capital area of Helsinki in Finland with at least one COVID-19 PCR confirmed case and at least one additional person living in the household. Respondents included children defined as over the age of 12, youth ages 13 to 17 and adults over the age of 18. Any number of household members could participate in the study.

### Recruitment

The study was part of a larger COVID-19 household transmission protocol that explored the extent of household transmission [37]. Individuals who were recruited for the transmission study, which included a series of household visits to test for the virus and antibodies, could also participate in the qualitative interview study during the final household visit.

Recruitment took place through the Finnish Institute for Health and Welfare website or via an SMS message that was sent to PCR confirmed cases in the capital area. The study coordinator contacted those interested in joining the study by phone to assess eligibility and to schedule a series of home visits. A qualitative interview was conducted 28+ days after the household index’s PCR confirmation when they were expected to have recovered from the virus. To protect the interviewer from potential transmission of the virus, a phone call was made to each household before the visit to ensure household members were free of symptoms. All but one interview was conducted in Finnish.

### Sampling

The sampling was based on maximum variation in which the overarching principle was to engage respondents from different types of households including couples without children, families with children, and families with teens so that their aggregate answers could be considered to reflect those of close to the whole population [38]. Interviews were conducted until no new information was generated and accordingly data saturation was reached [39].

### Conceptual framework and tools

The conceptual framework of health stigma informed the development of the stigma question guide. It included drivers and facilitators, manifestations, outcomes, and impacts of stigma. The drivers and facilitators are factors that encourage stigma. Drivers are conceptualized as inherently negative. Conversely, facilitators may be positive or negative influences. Drivers and facilitators determine whether stigma “marking” occurs, meaning stigma is applied to people or groups. Once a stigma is applied, it manifests in a range of stigma experiences and practices [25]. Stigma manifestations influence a number of outcomes for affected populations as well as for organizations and institutions, which then together influence longer term impacts of stigma. The study explored “self-stigma,” which is defined as a stigmatized group member’s own adoption of negative societal beliefs and feelings associated with their health status [25]. In this study, self-stigma explored negative feeling towards self among confirmed coronavirus individuals and their household members. The study also explored perceived stigma, which are the perceptions of a stigmatized group regarding how people treat them. In this study, perceived stigma explored how laboratory confirmed coronavirus cases and their household members perceived treatment by others. This framework was selected as it is not only theoretical, but facilitates an understanding of the factors that facilitate and mediate the stigmatization process for individuals and also informs intervention development [25]. Participants were asked to describe any negative experiences with people during their quarantine or isolation to record their experiences of stigma. The question guides also included a set of open-ended questions that explored their experiences in quarantine or isolation, such as: Tell me about your time in quarantine? How did quarantine influence your life? What was difficult for you during quarantine?.

### Interviews

Interviews ranged from 20 to 60 minutes and were conducted in a private room or space in the homes of the respondents. Interviews were conducted individually to allow for the exchange of sensitive or confidential information. All interviews were conducted in Finnish except one that was conducted in English. They were audio recorded and transcribed. Approximately 5% of completed interview transcripts (n = 3) were cross checked by another transcriber to ensure accuracy and the level of detail.

### Analysis

The analysis was based on a thematic analysis conducted by the first author (AL) [40]. The analysis of stigma followed the health stigma framework [25] that included identifying codes and categories within each construct of the framework (drivers, facilitators, manifestations, outcomes, and impacts of stigma). Analysis of the quarantine experiences were based on an inductive coding process.

The process started with a data familiarization process during which the analyst read the transcripts multiple times to get an overall idea of the dataset and to create an initial set of codes that resulted in a codebook. Coding was conducted for each interview using the codebooks, emerging new codes were also included using NVIVO12, followed by refining and expanding codes and developing categories. The initial analysis was shared with the study team (HN, TD, and LH) to get consensus on the emerging categories and the ways to explore relationships and patterns across the interviews. In the final stage, the analyst developed the interpretation. The syntheses of the results served as the foundation for operational recommendations to support people in quarantine and isolation.

### Ethics

The Finnish Communicable Diseases Law and the law on the duties of the Finnish Institute for Health and Welfare allowed the implementation of this research without seeking further institutional ethical review. Written informed consent was obtained from all cases and contacts willing to participate in the investigation, before each interview. A written consent for children under the legal age of consent (15 years) was obtained from a parent or legal guardian as well as from children under the age of 15.

## Results

### Participant characteristics

The study included 64 participants from 24 households in the capital area of Helsinki in Finland. Each household included at least one PCR-confirmed SARS-CoV-2 positive individual and 1–4 family members. Almost half of the households (42%) had children (0–12 years old), one-quarter of the families (25%) had teenagers (13–17 years old), one household was a single parent household, and the remaining households (29%) were couples without children or households where children no longer lived with their parents. The sample included 14 teenagers or children and four households included healthcare workers (nurses or doctors).

Most adult participants were in the age range of 30–49 (75%). The sample included approximately an equal number of female and male respondents. The PCR confirmed cases occurred in households during February-April 2020. The majority were confirmed in week 11 (50%) and week 12 (34%), while the remaining cases were confirmed in week 9, week 10 and week 13. Among PCR confirmed cases there was one teenager and three children who were confirmed positive. Household members who did not have symptoms were not PCR tested.

### Perceived stigma

**Fig 1:**
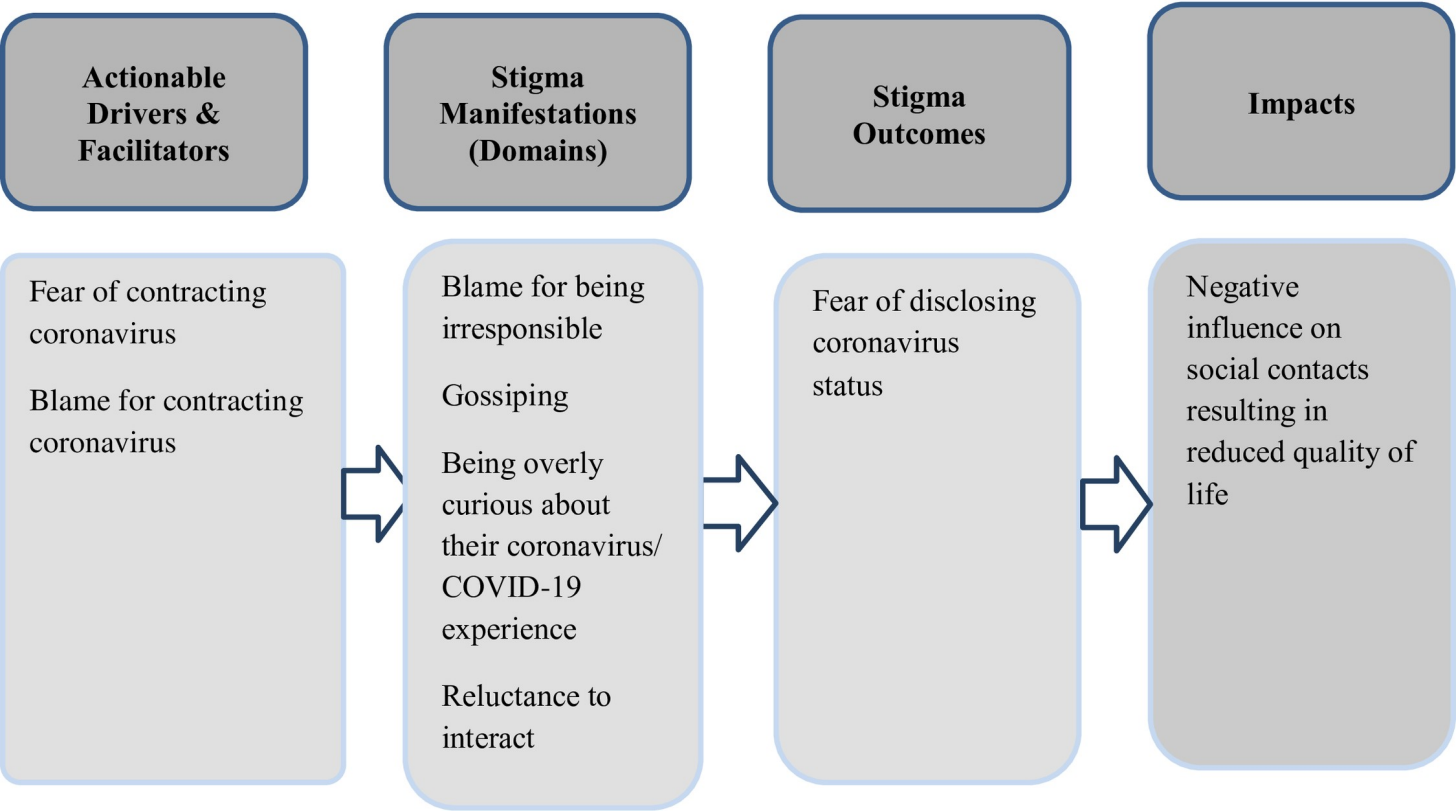
Framework for perceived stigma.

This section describes drivers, manifestations, outcomes and impact of perceived stigma. See Fig 1 Framework for perceived stigma. Most adult respondents had experienced stigma, while stigmatizing experiences among children and teenagers were less common. All stigma experiences of teen and child respondents included parent involvement, such as informing their friends or their families about their coronavirus status on behalf of the child or teenager. Respondents explained that most experiences of stigma occurred after quarantine because during quarantine their social contacts were minimal and accordingly there were few opportunities for stigmatizing experiences. Perceived stigma did not differ between respondents who experienced the infection during different periods of time nor between people who had wide social support networks or narrow social networks with whom to communicate. Stigmatizing experiences did not vary between respondents from different types of families or between those who were COVID-19 PCR confirmed cases, those who tested negative and those did not know of their coronavirus status, or between those with severe symptoms and those with mild symptoms, or among different types of families.

Those who did not report perceived stigma explained they had not thought of coronavirus as something stigmatizing. They included households with teens and children and in many of these households the infected individuals had experienced only mild symptoms.

### Drivers of perceived stigma

Respondents believed that they were stigmatized because of fear of getting infected with SARS-CoV-2 or because they were blamed for contracting the virus. Some respondents also believed that people were angry with them for putting others, especially children, at risk of infection.

A number of respondents highlighted that fear of contracting the virus was prominent because people were unsure how long individuals stayed infectious and whether the infection was followed by immunity and if so, for how long.

Healthcare workers experienced particular blame for contracting the virus.

“People think that we should not have any other life but being nurses or doctors. We should not be allowed to go out and contract the virus.” (Female healthcare worker respondent from a family with teenagers)

### Perpetrators and manifestations of perceived stigma

Respondents identified four types of stigma perpetrators linked with different types of social networks. The most distant social network of stigma perpetrators included those who had no personal relationship with the respondents but who communicated by posting comments on social media and traditional media sites. Stigma manifested in blame for being irresponsible and careless.

“After talking to media I got so many negative comments. I could not believe the reactions of people. They blamed us for being reckless. It felt really bad. I decided not to speak up again.” (Female respondent from a family with children)

The second network of stigma perpetrators were those who had an indirect link to the social networks of the respondents such as being parents at the same school as the respondents’ children or living in the same neighborhood, but not having a personal relationship with respondents. They stigmatized respondents by blaming them for contracting the virus, gossiping, and by being overly interested in their coronavirus and COVID-19 experiences.

“Yes we heard through friends that some people were accusing us of not informing everyone in the neighborhood about our infection. I am very sensitive about this kind of gossiping.” (Female respondent from a family with children)

The third network of stigma perpetrators included friends, acquaintances and colleagues with whom participants had not been in close contact before contracting the virus. Stigma manifested in the reluctance to interact with the respondents.

“There are many friends who are very straight forward that they won’t meet with us even outdoors.” (Male respondent from a family with children)

The fourth networks of stigma perpetrators were those who had been in close contact with the respondents before or during quarantine or isolation. Stigma manifested in being overly curious and continuously questioning them about coronavirus, the modes of transmission, and the ease of the transmission.

“It was quite obvious that they (friends) were afraid of my infection. It was tiring to answer the same questions over and over again. They also wanted us to give them instructions how to protect others from the infection and they did not believe that we had not received any such instructions.” (Male respondent from family with no children)

### Outcomes of perceived stigma

Stigmatizing experiences resulted in respondents’ reluctance to disclose their coronavirus status. Respondents frequently explained that disclosing their status to those that they had exposed was seen as a duty, but they disclosed to others only on a “need to know basis”. Some respondents explained they did not hide their coronavirus status from others, but only talked about it if asked.

“I still carefully consider every time I am in a situation in which I should tell about my COVID-19 status.” (Female respondent from a family with teenagers)

Outcomes of stigma among teenagers and children included insecurity about how to deal with friends and worry about the reaction of colleagues and friends at school.

“I am bit nervous about going to school. I know that people know we had coronavirus at home.” (Teenage respondent)

### Impact of perceived stigma

Respondents highlighted that perceived stigma negatively impacted their social contacts in many ways and accordingly reduced their quality of life.

“My life is not the same. There was more going on and more people around. It feels empty now.” (A female respondent from a family with children)

### Self-stigma

**Fig 2:**
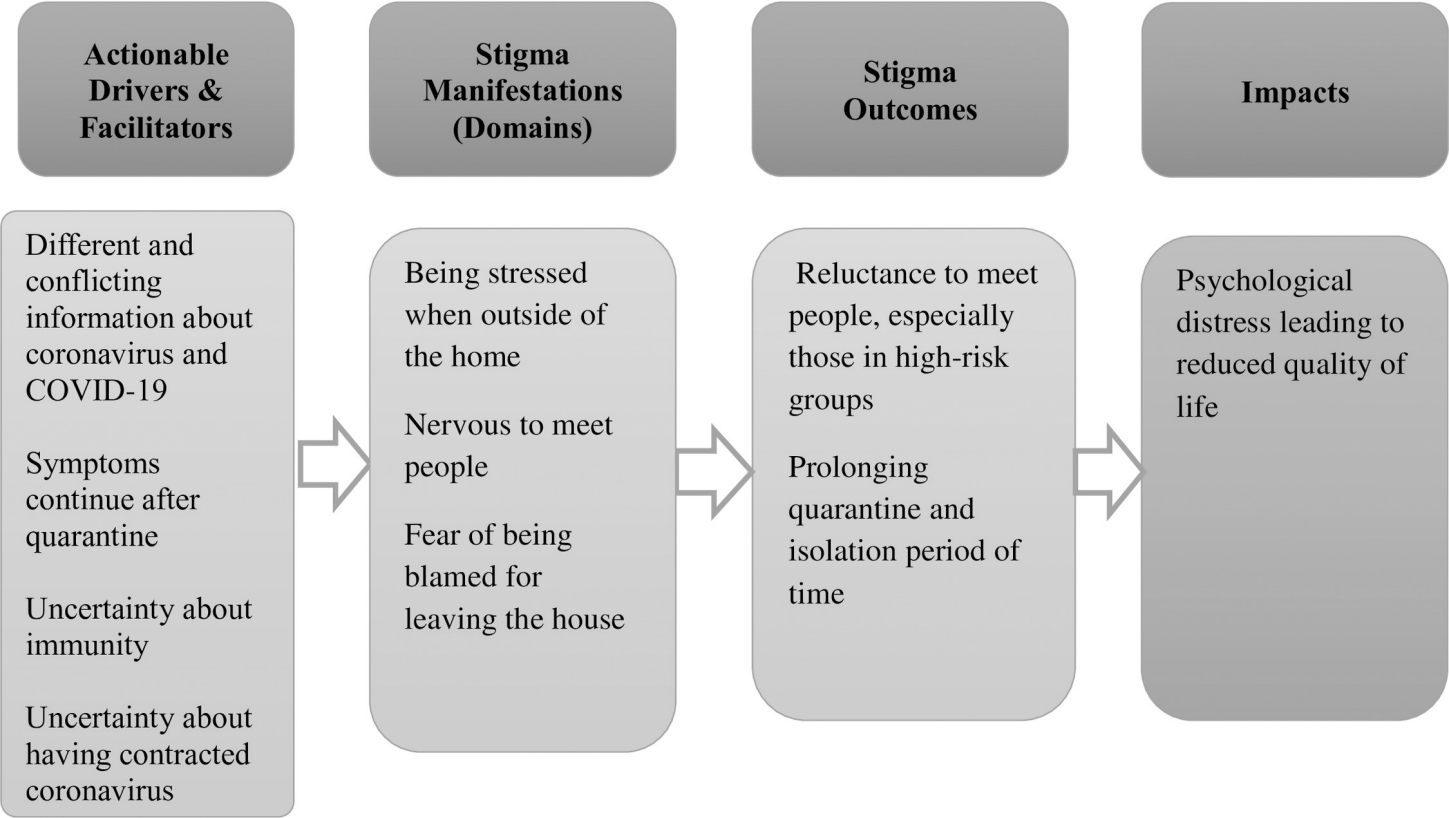
Framework for self-stigma.

This section describes drivers, manifestations, outcomes and impacts of self- stigma. See Fig 2 Framework for self-stigma. Participants were asked to explain the kind of negative feelings they had towards self to better understand how they defined self-stigma. Participants explained that self-stigma meant feelings of being contagious or infectious and accordingly worry about infecting others, which was particularly common after the quarantine or isolation ended.

Almost all adults had negative feelings towards self, whereas none of the children, youth or healthcare workers or their family members expressed self-stigma. Self-stigma did not differ between respondents experiencing the coronavirus during different periods of time nor between people who had wide social support networks and those who only had a few people with whom to communicate. Self-stigma did not vary between respondents from different types of families, between or between those who were COVID-19 PCR confirmed cases, those who tested negative and those did not know of their coronavirus status, or between those with severe symptoms and those with mild symptoms.

### Drivers of self-stigma

Respondents highlighted that negative feeling towards self were driven by limited and changing information about the virus as well as limited and often contradicting information about quarantine procedures. Respondents also highlighted that information about quarantine did not include many concrete or practical instructions, which led to uncertainty about how to manage daily life during quarantine or isolation and how not to infect others. Some respondents worried that the health authorities had ended their quarantine time only because there were not enough workers to follow up with all positive coronavirus cases. Similarly, some COVID-19 PCR positive cases worried about being potentially infectious as their quarantine ended while they were still symptomatic. In addition, respondents expressed insecurity surrounding immunity as yet another reason that kept them worried about potentially still being infectious.

“I had hoped that once I was done with quarantine that my life would return to normal but it did not. I continued to be worried about transmitting the infection to others.” (Female respondent from a family without children)

Some respondents who did not know about their coronavirus status often struggled for several weeks to get PCR-tested for SARS-CoV-2 during which continuous uncertainty played a central role in their lives. The same uncertainty continued after quarantine.

“I do not know what I should think about myself. Did I have the infection [COVID-19] or not?” (Male respondent from a family with teenagers)

### Manifestations of self-stigma

Adult respondents explained that self-stigma created stress when leaving the house and when in close proximity to others in shops, markets and even outdoor areas such as in parks. Meeting friends in the street and greeting people made some respondents tense; some worried about being blamed by others for being outside of their home. COVID-19 PCR respondents expressed greater stress about encounters with people outside of the house than others.

“I was thinking that neighbors are watching and wondering why I’m going to do the shopping.” (Female respondents from a family with children)

Healthcare workers and their family members did not express fears about being infectious after quarantine. On the contrary, they were confident about having immunity that protects them and others.

“It may come back [COVID-19] but I would be really surprised if no antibodies were detected in me.” (Female respondent from a family with a healthcare worker)

Children and teenage respondents did not explain situations in which they felt difficulty interacting with people outside their house. On the contrary, some of them claimed having breached quarantine to meet friends.

“My sister told me not to go out but it was so frustrating to stay at home. I really missed my friends. So I met my girlfriend outside of the house a couple of times.” (Male teenage respondent)

### Outcomes of self-stigma

Adult respondents explained that self-stigma made them reluctant to meet people outside of their household, and in particular, with people who belonged to high-risk groups. Many of them expressed being devastated about not being able to meet elderly or sick parents in person and not knowing when they would able to do so.

“I know I am no longer sick but I haven’t been able to meet with my parents. I really do not dare to do that. This is a real tragedy for us, but I am too worried about infecting them.” (Male respondent from a family with children)

Adult respondents also prolonged their quarantine after the official quarantine time had ended by an additional one to even three weeks because of fear of infecting others

“We just wanted to be on the safe side and continue our quarantine for an additional two weeks.” (Female respondent from a couple without children)

Two adult respondents expressed guilt about having infected people outside of their household who belonged to high-risk groups. Respondents did not express guilt about infecting members of their own household, which they saw as inevitable.

“I felt so guilty for having infected my mother. I visited her every day. My isolation was all about worrying about my mother.” (Female respondent from a family with children)

### Impact of self-stigma

In general, respondents agreed that self-stigma was a burden that created psychological stress.

“These negative feelings that I have towards myself are creating so much tension and stress in my life.” (Male respondent from a couple without children)

### Coping mechanisms to manage stigma

Respondents explained they managed stigma in multiple ways. Some of them felt that the best way to avoid stigma was to challenge the fear of being stigmatized by telling everyone with whom they had been in contact about their coronavirus status. However, not all respondents could overcome the fear of stigma to do so.

“We told everyone so nobody will come later to blame us.” (Male respondent from a family with children)

Others explained that they avoided stigma by following the instructions for isolation or quarantine and ensured that everyone who knew about their coronavirus infection knew that they were doing the right thing to prevent the transmission of the virus.

“We were always at home as they requested us to do. This was also a way to avoid conflicts and reduce the fear people had towards us.” (Female respondent from a family without children)

Some respondents reduced self-stigma by convincing themselves that they had done everything they could have to avoid contracting coronavirus. Others believed that increasing quarantine time by an additional week or two reduced self-stigma.

“We took decisions based on the available information. There was nothing more we could have done at that time to avoid the infection.” (Female respondent from a family with children)

### Quarantine and isolation experiences

This section describes key characteristics linked with quarantine experiences (worry about health and boredom) as well as communication and everyday routines during quarantine and isolation.

### Worries about health

Respondents from households with severe or prolonged coronavirus-related symptoms or from households where household members had difficulty accessing testing or being admitted to the hospital often described their quarantine experience as having revolved around symptom management and worry about escalating symptoms and even death. For some respondents this was an overwhelming experience. Respondents explained thinking about death in particular when a friend or someone they knew was hospitalized or when media reported about high infection rates and fatalities abroad. Respondents explained that they passed the time by analyzing their changing symptoms.

“I was living in continuous worry and fear. No one was able to explain how the illness will develop.” (Female respondent from a family with children)

Some respondents found themselves caring for their sick household members, which consumed all of their time and energy. Many of them felt incapable of the task and therefore considered it a heavy responsibility. Some respondents felt sorry for their children who were ignored and forgotten while they managed the illness among other household members.

“My life was revolving around my sick husband. I could not rest or think; I just kept going from one day to the next. I wanted him to be admitted to hospital as I did not want to carry all that responsibility myself.” (Female respondent from a family with children)

Children and teen respondents explained being worried when seeing one or both parents severely ill. Some of them worried about the condition of their parents and what they could do to help the situation, others were more worried about practical issues such as what to do if both parents ended up in the hospital. Neither children nor teenagers seemed worried about their own health.

“I was scared when both of my parents were sick. I went through an emotional rollercoaster when my parents were ill.” (A male teenager respondent)

Some respondents were consumed with worry about the people they exposed to the coronavirus or who contracted the virus from them.

“I had no time to think about myself or worry; I was so devastated about my mother. I felt so guilty for infecting her.” (Female respondent from a family with children)

Others worried about friends and family members in high-risk groups who may get sick or about not being able to socialize with them.

“We haven’t met with my parents since this all started. I am so worried and we are all so sad. I kept thinking of my parents a lot.” (Female respondent from a family with no children)

### Boredom

Some respondents from households with mild COVID-19 symptoms described quarantine as monotonous with the same daily routines. Some were irritated about their everyday lives. Teen and child respondents also frequently described quarantine as tiresome and dull. One of them explained that quarantine made him and his friends extremely passive. Respondents who used to meet with friends and had an outgoing and active lifestyle were particularly bothered about the restricted lifestyle in quarantine.

“It is exhausting to be at home, all days are the same. Although I manage to work from home just fine, the days are the same.” (Male respondent from a family without children)

Others with mild symptoms did not consider that their everyday life had changed much since they contracted the virus. They explained spending a lot of time at home, working from home, or having little social contact outside of their home.

“Our life is already like quarantine so this situation has brought us no major changes for us.” (Male respondents from a family with teenage children)

### Communication

Over half of the participants had at least one person in the household who was communicating regularly with others who had contracted the virus. The most common communication channel was a WhatsApp group that was established among people who attended an event during which they contracted the virus or a pre-existing WhatsApp group where friends who had contracted coronavirus were already communicating.

*“I don’t know what I would have done without my group*. *I was talking to them daily*. *It helped me to ensure that I was going to manage this*.*”* (*Female respondent from a couple without children)*

Those who did not belong to such peer support groups included participants who did not know the mode of transmission of their infection or those who were elderly.

Respondents explained that regular communication with others, including listening to the illness episodes of others and finding similarities with their own experiences, reduced worry about their own health and the health of other coronavirus positive household members. Communication with peers also provided an opportunity to share their own experience with those who understood their situation. Respondents said that being part of a group empowered them: “Together we can make it.” In addition, respondents mentioned that groups provided information about how to access testing and they helped them prepare for being positive for SARS-CoV-2. Communicating with others in the same situation was also important to healthcare workers.

For some respondents participation in coronavirus-specific communication groups was a negative experience as continuous discussions about the topic made the illness highly present in their lives and a source of stress. One respondent felt that the active WhatsApp communication by his partner made him an outsider. For other respondents, active WhatsApp communication with other household members was a great source of information for the whole household.

“It was a bit too much for after a while with all the information I received from the group. I felt that there was nothing else in my life than corona so I started distance myself from the group.” (Female respondent from a family with children

Some respondents discussed their infection in other personal WhatsApp groups to share coronavirus and COVID-19 information with others or to get attention. Some respondents explained that they had the role of “corona experts” in those groups whose advice friends sought through the group.

“My friends who are worried about corona keep consulting me about their symptoms. I turned out to be corona expert.” (Male respondent from a family with children)

Teen and child respondents did not explain having established any coronavirus-specific groups. Some of them communicated with friends from time to time about their infection, but more often their discussions related to quarantine and the problems that it brought to their lives such as cancelled graduation parties, sports and travel.

“We used to talk about corona in the beginning with friends but not so much anymore. Now we wonder when all this will end and we get back to our lives and our activities.” (A male teenage respondent)

Many respondents highlighted that they were cautious talking about worries and fears related to coronavirus with their own family members to avoid elevating problems. Some respondents clarified that talking about the infection might increase their fears or generate more serious situations.

“I did not want to open up discussion about my fears with my family. They were worried enough. I thought it may upset them more.” (Female respondent from a family with teenagers)

Respondents explained that media coverage was exhausting and stressful and some of them had stopped actively following it. Other participants claimed following all news related to coronavirus on a daily basis. Several adolescent respondents felt that the media coverage irritated them and accordingly they no longer followed the coronavirus coverage regularly.

“I stopped following news about coronavirus some time ago. It was making me depressed.” (A female teenage respondent)

Some respondents mentioned that following media coverage had been helpful as it allowed them to prepare themselves for the possibility of contracting the coronavirus.

“I was not surprised about it. I knew what to expect. I had heard so much about it over the past weeks.” (Female respondent from a family with teenagers)

### Everyday routines

Not many respondents took precautions with their living arrangements to protect others in the household from coronavirus. Some considered their house too small to take any special precautions, others felt it unnecessary because they discovered the infection late and accordingly had been living together without precautions for a while. A few respondents had not thought of such precautions or they explained not having received any such instructions from the health authorities. Those who organized their everyday life to avoid transmission of the virus within the household slept in different rooms, lived on different floors or in other areas of the house, used separate kitchen utensils, separate toilets and bathroom, or undertook more intensive cleaning procedures.

Leaving the house during quarantine varied from those who did not leave their house the entire time to those who took short walks and those who took a regular walks with their dog or children on a daily basis. Some participants were far too sick to leave their house, and others were unsure about what they were allowed to do and discussions in WhatsApp groups confused them further.

”They said we can go to the backyard. But we do not have a backyard. We live in an apartment building in the middle of the city.” (Female respondent from a couple without children)

### Family relations

Some respondents believed that quarantine created tension at home between the household members. One respondent explained her child was having constant tantrums due to the shift from an active lifestyle to being at home. But several respondents saw some positive developments from quarantine, such as getting closer to their partner, having more time as a family, or time to relax.

“We have gotten closer one another due to this experience that we shared.” (Male respondent from a family with no children)

## Discussion

To our knowledge, this is the first study in Finland exploring COVID-19-related social stigma and the quarantine and isolation experiences. The study indicated that social stigma poses a challenge by impacting social relations and by creating psychological distress, which is likely to affect quality of life particularly after the illness. Quarantine and isolation experiences were characterized by worry about health and boredom. Communicating with others who contracted coronavirus was common, but fears and worries were only cautiously communicated to family members. Living arrangements did not include specific precautions to prevent further transmission at home. Activities outside the home during quarantine were limited but varied.

The study findings indicated that respondents did not feel a sense of closure after their isolation and quarantine had ended because of perceived stigma and self-stigma, and worry that they could still infect people around them [19–41]. Stigma reduced their willingness to disclose their coronavirus status or any household association with it leading to psychological and social distress. Stigma is known to result in delayed health seeking behavior among symptomatic patients including testing which can speed the transmission of the virus rapidly [42, 43]. This can be particularly problematic in countries where the focus of the national COVID-19 prevention strategy is testing and contact tracing followed by isolation and quarantine such as currently in Finland.

The study findings also indicated that the way adults communicated about coronavirus in the social networks of their children created stigma. The public and, in particular, parents will benefit from guidance on how to communicate about coronavirus and COVID-19-related issues. Marketing of de-stigmatizing wording has been identified as helpful to reduce stigma elsewhere [44, 45]. In addition, stigma has been successfully reduced by communicating the rationale behind potentially stigmatizing concepts of quarantine, isolation or suspect cases [11].

Fear and blame were drivers of coronavirus and COVID-19 stigma, which are also common drivers of infectious disease stigma worldwide [46–48]. In today’s world, fear is easily generated by an overabundance of news, mixing facts, rumors, and fake news [49]. Misinformation about COVID-19 has also rapidly spread around the world through social media [50]. Accordingly, the provision of factual information about transmission and the nature of the virus have been found to be an effective strategy to reduce stigma [43, 44, 51]. Stigma reducing information should include expert information about the disease such as contagiousness, number of diagnosed people, fatality rate, and seroprevalence in the community. In addition, the information should also provide people with tools about how to prevent the infection to ensure that people feel confident about being able to protect themselves, which in turn will prompt their willingness to apply protective measures [43]. New and innovative ways to communicate about SARS-CoV-2 and COVID-19 should be considered [52].

Overall, provision of accurate information about COVID-19 is challenging as information is rapidly evolving. Therefore, health authorities should focus on ensuring that the public is aware of trustworthy and updated sources of information where they can look up continuously changing information about the virus as a means to help build public confidence to manage better their interactions with those who contracted the virus. Having a trusted source provide information to the public is crucial as demonstrated in an anti-stigma intervention in Sicily where trusted health experts engaged themselves with the public through an online platform and successfully reduced COVID-19-related stigma [43].

Blame for infection could be reduced through messaging that anyone can contract the virus and anyone is vulnerable [44]. Blame could be reduced by using emotions in the communication though symbols or metaphors [53]. Special attention should be given to address blame for the infection that is directed towards healthcare workers who are already likely to be under high pressure. Stigma towards healthcare workers caring for COVID-19 patients has been identified in a number of countries worldwide [31]. In particular, actions are needed to promote their well-being during and after the outbreak. The UK has developed a digital learning package for healthcare workers’ well-being during and after COVID-19 that might be worthwhile to explore in Finland and in other settings [54].

As both traditional media and social media promoted stigmatizing attitudes. Stigma capacity building training could be organized for media representatives and social media influencers, such as raising awareness about how to recognize stigmatized groups and how stigmatization is enacted. The content of anti-stigma training often includes information about the ways to identify stigmatized persons, about responsibility for their condition, about the moral and physical peril linked to them, as well as labels for the stigmatized group [55].

The study discovered that perpetrators of stigma belonged to different types of social networks ranging from close friends to those who had no personal relationship with the respondents. Likely, those who stigmatize persons with coronavirus and their family members do not necessarily understand the harm they cause through their actions and words. Messages of empathy could be promoted to develop a more sensitive and caring atmosphere for those with coronavirus and their household members. Stigma reduction efforts that focus on promoting emotional approaches could be used as guidance such as contact interventions [52, 55]. At the same time, those with coronavirus and their family members need to be empowered to manage dealings among the social networks of those with coronavirus during and after quarantine. Children and teenagers could be provided with special “back to school instructions” to reduce the worry about how to meet friends at school. Guidance about how to deal with members of high-risk groups after quarantine would be important as well.

Due to changing and contradictory information received during the epidemic, uncertainty was central in the experiences of those quarantined and isolated as was similarly identified in recent study in China [11]. This highlights the need for a national communication strategy under which all authorities and academic institutions communicate at all levels to ensure that that all health personal disseminate the same information in the same way [56].

The findings showed that health officials left out a number of important audiences in communications about the virus. Firstly, those who cared for sick household members were usually not in touch with health officials. This left caretakers without critical information and opportunities to discuss and get advice. Many caretakers were overly stressed about the responsibility they were left to manage. Secondly, children and teenagers did not have a specific channel to communicate with health officials to gain information or share fears and concerns. Thirdly, asymptomatic household members or those who tested negative received less attention although they seemed to have equally pressing uncertainties compared to others. A recent study in China concluded that the close contacts of those with coronavirus suffered from physical and psychological problems that must be addressed [11]. There is a need to explore how to engage all household members in the communication with health authorities who follow up and provide assistance for families with coronavirus. Models for expanding communication to families instead of patients could be taken from countries that have a system of family doctors where communication is often also family based [57]. Digital platforms could be considered to reach all household members.

Individual behavior is of central importance to control the spread of coronavirus [32]. The study showed that quarantine and isolation may be accepted interventions among the public in Finland. Although boredom among more social and active participants during quarantine and isolation was prevalent, there were also those who did not perceive quarantine particularly different from their everyday life as reported also in Canada [58]. Moreover, participants used physical activities as coping mechanisms to manage the boredom such as walking and jogging similar to a study in China that showed that quarantined individuals focused on physical health to manage the situation [11].

Communicating with other people with coronavirus via WhatsApp developed naturally based on the current communication culture in which WhatsApp groups are based on themes and or social networks and are a major channel to communicate with others [59]. Respondents noted that finding others in the same situation and having the opportunity to discuss the infection and related developments were of utmost importance as identified in a number of studies elsewhere [60]. Communicating with others with coronavirus provided information and reduced worries, however, communicating with peers did not reduce self-stigma or uncertainties around coronavirus and COVID-19. On the contrary, sometimes discussions in peer WhatsApp groups confused the situation further. Moreover, WhatsApp groups have also been identified as major channels of misinformation [61]. Communicating about coronavirus and COVID-19 within families was more problematic as household members feared they would elevate the problem by talking about it. Emotional support group communication for children and youth has been identified as a way to help participants express sorrow and worry [62].

There are limitations in our study that must be acknowledged. Sampling following maximum variation was fulfilled in terms of engaging different types of households. However, variation regarding the age group of participants and the time of onset of illness had only limited variation which may have been influenced by the mobile and web-based recruitment process that may have excluded elderly participants who are potentially less likely to communicate through those channels or less comfortable using the devices. In addition, the perceptions reported in interviews might have been influenced by social desirability bias given the potentially sensitive nature of topics such as feelings and family dynamics.

## Conclusion

This study provided valuable information about how to support those in quarantine and isolation, which can be used to ensure health interventions are acceptable to the public. It resulted in a set of recommendations to reduce stigma including promotion of destigmatizing language, addressing fear of infection by promoting reliable sources of COVID-19 information, and addressing the blame of infection through messages that reinforce ideas that “anyone can get infected.” In addition, recommendations include conducting stigma trainings for media and social media influencers, using emotions as a communication approach, empowering those with coronavirus and COVID-19 and their family members to manage stigma, and advocating for the creation of a national communication plan to ensure aligned messaging about coronavirus and COVID-19 across the country.

To better manage quarantine and isolation, the study recommended ensuring the delivery of sufficient and practical information about coronavirus, COVID-19 and quarantine to all household members including children, teenagers, asymptomatic and SARS-CoV-2 negative individuals. The information should address practical matters related to everyday life and how to stay active in quarantine and isolation.

## Supporting information

S1 Dataset (DOCX)

S1 Question guide (DOCX)
