# Supplementary material for: Learning about COVID-19-related stigma, quarantine and isolation experiences in Finland
Source: PLoS One. 2021 Apr 14;16(4):e0247962. doi: 10.1371/journal.pone.0247962 (PMC8046198; doi:10.1371/journal.pone.0247962)
Supplement: S1 Dataset — (DOCX) [file pone.0247962.s001.docx]

Koronakoti

Nodes

| Name | Description | Files | References |
| --- | --- | --- | --- |
| 27 ajatellaan että ollaan sotatlassa jollin usko auktoriteetteihin kasvaa |  | 1 | 1 |
| 27 ajatusta korona uhasta kasvoi ja enne sairatsumista puhuttiin jo korona ajasta |  | 1 | 1 |
| 27 coping en voinut asialle mitään |  | 1 | 1 |
| 27 Coping mechanism että huolehdin muista enkä itsestäni |  | 1 | 1 |
| 27 coping yrottää olla ajattelematta |  | 1 | 1 |
| 27 ei asunijärjestelyjä koska asunto on liian pieni |  | 1 | 1 |
| 27 ei syyttelyä koska konsertin aikaan ei voinut tietää ja kaikki tejtiin laillisesti |  | 1 | 1 |
| 27 ennen tetsituloksia selvittelee käytännön asioita karanteenia varten |  | 1 | 2 |
| 27 hengenvaarallinen mahdollisesti |  | 1 | 2 |
| 27 huloli että lasnettarha ei huoli lasta |  | 1 | 1 |
| 27 huoli puhkesi empatiaksi |  | 1 | 2 |
| APUA |  | 0 | 0 |
| Fudis ryhmän Whats App auttoi koordinoinnissa median kanssa |  | 1 | 1 |
| Koulu ja HJK otti viestinnän niskoilleen |  | 1 | 1 |
| Taloyhtiön FB sivulla kyselin jos joku voi auttaa ruokaostoksissa |  | 1 | 1 |
| Vaikea saada aikaa lääkärille jos ei valehtele omia oireitaan |  | 1 | 1 |
| Vaikeaa päästä testeihin |  | 1 | 5 |
| ASUMINEN |  | 0 | 0 |
| Asuttiin erillämme |  | 1 | 1 |
| Ei liikuttu edes omassa pihapiirissä |  | 1 | 1 |
| Ei ollut kotivarotoimia ajateltiin että kun ollaan samassa talossa niin kaikki sen saa |  | 1 | 3 |
| Ei oo paikkaa missä näkis äijiä |  | 1 | 1 |
| Elettiin päivä kerrallaan |  | 0 | 0 |
| Elämä ei ole kauheasti muuttunut |  | 1 | 1 |
| En pajokaan tiennut covidista ennen kuin tuli omalle kohdalle |  | 1 | 1 |
| Erikoisjärjetelyä kotona sen veraan että makaisn sohvalla joka oli vähän erilään muista |  | 1 | 1 |
| Jos rajoituksia jatketaan pitkään niin ihmiset ei jaksa |  | 1 | 1 |
| Jännää aikaa kaikille |  | 1 | 1 |
| Kaipasin ulos mutta viihdyn sisällä |  | 1 | 1 |
| karanteeni oli rankaa koska oltiin äidin kanssa erillään toisistamme |  | 1 | 1 |
| Karanteeni on menny huippukivasti |  | 1 | 2 |
| Karanteeni on ollut kuin jatkettu äitiysloma |  | 1 | 1 |
| Karanteeniaika oli tylsää |  | 1 | 1 |
| Karanteeniaika on ollut tylsää ja tehotonta mutta ei dramaattista |  | 1 | 1 |
| Karanteenin ajan olen aina käynyt ulkoiluttamassa koiria |  | 1 | 1 |
| Karanteenin loppuvaiheessa kaipasi ulos ja liikkumaan |  | 1 | 2 |
| karanteenissa oli ikävintä että ei nahnyt kavereita ja piti olla koko ajan perheen kanssa |  | 1 | 1 |
| Kaverit sano että tulee tylsää |  | 1 | 1 |
| Kävimme eristyksenkin aikana kävelemässä ulkona missä ei ole ihmisiä |  | 1 | 1 |
| Masennuuttaa kun ei tiedä koska tämä loppuu |  | 1 | 1 |
| Me ajateltiin että me ei voida likkua kun asutaan keskellä kaupunkia |  | 1 | 1 |
| Mieheni jolla oli koronatartunta ei koskenut vauvaan |  | 1 | 1 |
| Miten liikkuminen meidän ja niiden välillä voidaan järjestää |  | 1 | 1 |
| Monet alkaa tulla ahdistuneiksi kotonaolemisesta |  | 1 | 1 |
| Oleskelttiin erillään äidin kanssa |  | 1 | 1 |
| Olin aika karanteenissa näin mm tyttöystävääni |  | 1 | 1 |
| Oma ympäristö on suhtautunut rationalisti mutta on muilta kuullut juttuja että deisfoidaan kaikki ruokostokset |  | 1 | 1 |
| On kahta eri koulukuntaa toisia jotka ovat totaalisesti eistäytyneet ja toisia jotka ottavat rennommin |  | 1 | 1 |
| Outoa että ei näe ketään |  | 1 | 2 |
| Parhaat ystävät sairatsuivat yhtä aikaa mikä oli jännittävää |  | 1 | 1 |
| pihapiirissä saa liikkua |  | 1 | 1 |
| Poistuttiin kotoa vain testeihin |  | 1 | 1 |
| Pääsykokeisiin lukeminen pikkukuumeessa ei ole helppoa |  | 1 | 1 |
| Ruoan kotiintilausta alettiin käyttää jo ennen koronaa |  | 1 | 1 |
| Ruoan tilaus netistä oli jo alettu kuukausi ennen koronaa |  | 1 | 2 |
| Ruoka tilattiin netistä |  | 1 | 1 |
| Rutiinit auttoivat selviämään |  | 1 | 1 |
| Sairastuneet eivät voi liikkua ulkona |  | 1 | 1 |
| Tein töitä |  | 1 | 1 |
| Töitten tekeminen kuumessa oli stressaavaa |  | 1 | 2 |
| Vaikeaa olla sisätiloissa neljän seinän sisällä |  | 1 | 1 |
| Vaikeinta ei näe kavereita |  | 1 | 2 |
| veljen kanssa riidellään samalla tavalla kuin ennen |  | 1 | 1 |
| Whats App ryhmän kanssa saatetaan katsoa samaa ohjelmaa yhdessä |  | 1 | 1 |
| Videopelien kautta keskustellaan samalla kavereitten kanssa |  | 1 | 1 |
| Coping peilaan makrotasoa |  | 1 | 1 |
| EPÄVARMUUS |  | 0 | 0 |
| Aina mainitsin raskauden |  | 1 | 1 |
| Ajattelin että eristys purettiin liian aikaisin kun ne ei jaksaneet enää soitella minulle |  | 1 | 1 |
| Aluksi uskoteltiin että influenssa vaikka oireet alkoivat viitata vahvasti koronaan |  | 1 | 1 |
| Auttaisi yhteiskuntaa jos sanottais että voit mennä etkä tartuta ketään |  | 1 | 1 |
| Ei ole linjattu että taudin sairastaneet saavat liikkua normaalisti |  | 1 | 1 |
| Ei pelottanut mutta tuntui oudolta että on tällainen tilanne L |  | 1 | 1 |
| Ei tiedä miten kauan immuniteeti kestää |  | 1 | 1 |
| Ei voi luottaa poliitikkoihin kun ne puhuu eri tavalla joka päivä |  | 1 | 1 |
| En ole voinit sanoa että minulla on korona koska sitä ei le todettu |  | 1 | 1 |
| En pelkää |  | 0 | 0 |
| En ollut erityisen huolissani kun olen perusterve ihminen |  | 1 | 1 |
| Epoävarmuuttaa kaksi viikkoa on takana mutta perheenjäsenillä ei ole |  | 0 | 0 |
| Epäselvää aluksi että olivatko kaikki vieraat karanteenissa |  | 1 | 1 |
| Epäselvää kun vanhemmat toimii eri tavoin |  | 1 | 2 |
| Epäselvää mistä päivästä lasken että oireet on ohi |  | 1 | 1 |
| Epäselvää mitä voi tehdä kiun on oireellinen, kun on sairastanut |  | 1 | 1 |
| Epäselvää oli että mikä on ohejitsu jos lapsilla on exposure |  | 1 | 2 |
| Epävarmuus onko ollut korona |  | 0 | 0 |
| Epävarmuus siitä miten mennään eteenpäin oli hankalaa |  | 1 | 1 |
| Epävarmuutta miten toimia kun ei ole autoa ja muista tartuntariskeistä |  | 0 | 0 |
| Epävarmuuttaa koska on annettu ristiriitaisia ohjeita |  | 2 | 2 |
| Erilaista informaatiota |  | 0 | 0 |
| Eristys purettiin oli nuhaa joten ei tuntunut siltä että tauti oli päättynyt |  | 1 | 1 |
| Hanakalaa kun vanhemmilla on erilaisia linjoja |  | 1 | 1 |
| Ihmetyttää että en ole saanut koronaa ollenkaan (lapsi) |  | 0 | 0 |
| Ihmiset oli huolissaan että mitä se tarkoittaa heille |  | 1 | 1 |
| Inhottava tunne kunnes pääsen ultraan |  | 1 | 3 |
| Jatkoin itse karanteenia kun oli nuhaa |  | 1 | 1 |
| Karanteeni on loppunut mutta koska yhdellä lapsella on kuumetta niin ei tunnu että se on ohi |  | 0 | 0 |
| Karanteeni on sekaavaa |  | 1 | 2 |
| Karanteeniin jääminen oli epäselvää |  | 1 | 1 |
| Karanteenin päättymisen aikaraja on ollut ongelmallinen kun tyttärellä on edelleen kuumetta |  | 1 | 1 |
| Karanteeniohjeet muuttuivat kokoajan |  | 1 | 2 |
| Kun oireet tuli en halunnut mennä lentokoneeseen jos oireet olisikin koronaa |  | 1 | 1 |
| Lapset tarvisisvat tietoa |  | 1 | 1 |
| Lasten vanhempien keskuudessa on selvästi epäselvää mikä on turvallista ja mikä ei |  | 1 | 1 |
| Lähtisin isoihin tapahtumiin kyllä mulla on olo että tämä on nyt sairastettu |  | 1 | 1 |
| Mielenrauha täytyy saada itselle että tauti on koettu ja immuniteetti saatu (ideaali tilanne) |  | 1 | 1 |
| Mitä tarkoittaa saa kävellä ulkona |  | 0 | 0 |
| Mitä tarkoittaa saa liikkua |  | 1 | 1 |
| Ohjeitus oli hidasta joka nosti pelkoa että asia riistäytyy käsistä |  | 1 | 2 |
| Olen ihmetellyt että miten en ole saanut tartuntaa |  | 1 | 1 |
| Olin huolisaani kovien oireiden aiknaa mutta en halunnuyt huolestuttaa vaimoa |  | 1 | 2 |
| Oltiin eristyksissä kaksi viikkoa ja pari päivää päälle |  | 1 | 1 |
| On sanottu että karanteeni loppuu mutta käsketty olla varovaisia |  | 1 | 1 |
| Paljon kysymyksiä kun en ole tiennyt onko tämä koronaa |  | 1 | 1 |
| Pelotti tilasin ambulanssin joka loputa sanoi että ei tarpeeksi huonossa kunnossa joten eivät ottaneet sairaalaan |  | 1 | 1 |
| Pidin omaa tällaista vielä sen jälkeen |  | 1 | 1 |
| Pohdittu jälkeenpäin oliko meillä riittävästi tietoa |  | 1 | 1 |
| Pääsi sairaalaan pitkän suostuttelun jälkeen kun sanoin että en jaksa |  | 1 | 1 |
| Ristiriitaisia tietoja kuinka pitkä aika on karanteeni |  | 1 | 1 |
| Sairaana oli illuusio että sitten kun olen sairastanut että kaikki on ohi |  | 1 | 1 |
| Suomessa ei reagoitu tähän |  | 1 | 1 |
| Testauksessa on se hyvä asia että loppuu epätietoisuus |  | 1 | 1 |
| Tiedon muuttuminen ja epävarmuuss on hankalaa |  | 1 | 1 |
| Toisen informaation varassa |  | 1 | 1 |
| Vaikea saada apua joten ajettiin sairaalaan |  | 1 | 3 |
| Vaikea tukea miestä koska en tiennyt mitä ohjeita hänelle annettiin |  | 1 | 1 |
| Vaikeaa kun määräykset karanteenista perustuu omaan fiilikseen |  | 1 | 1 |
| Vapaaehtoinen kothoito kun minua ei kerran testattu vaan sanottiin että on flunssaa |  | 1 | 1 |
| Viranomaiset soitteli aina sairaalle miehelleni joka ei aina pysnyt perässä |  | 1 | 1 |
| Viranomaiset vain pumppasivat tietoja ei mitään empatiaa osoitettu |  | 1 | 1 |
| Viranomaisilta tuleva apu olis nopea testaus ja paikka mistä saa neuvoja |  | 1 | 1 |
| HAASTATTELU17 |  | 1 | 1 |
| Ambulanssi kieltäytyi kuljettamasta |  | 1 | 3 |
| Asuminen laöpset yläkerrassa |  | 1 | 2 |
| Asumisjärjestelyt |  | 1 | 1 |
| Ei sukallettu keskustella asiasta |  | 1 | 1 |
| Ei usko että leimaa tulee |  | 1 | 1 |
| Ei voi halata omia lapsia on kauheaa |  | 1 | 1 |
| En halua että syytellään joten pysyn kauempana |  | 1 | 1 |
| Epävarmutta kun ei päässyt testeihin |  | 1 | 4 |
| Huolissaan siitä että omat lapset ovat huolissaan |  | 0 | 0 |
| Hyvä että sairastettiin nyt |  | 1 | 1 |
| Konkreettisia neuvoja että miten eletään ja mikä voi tartuttaa. sama hammastahna |  | 1 | 1 |
| Lukenut paljon ja yrittänyt ymmärtää |  | 1 | 1 |
| Naapurit varoo |  | 1 | 1 |
| Olen kertonut kaikille töissä |  | 1 | 1 |
| Paniikissa kun näkee vanhuksia |  | 1 | 1 |
| Pelkoa mitä jos joudutaan molemmat yhtä aikaa sairaalaan |  | 1 | 1 |
| Vitsailtiin että onkohan koronaa |  | 1 | 1 |
| Välttelen edelleen työkavereita |  | 1 | 2 |
| Yllätys että tauti oli niin kova |  | 1 | 2 |
| HAASTATTELU21 |  | 0 | 0 |
| Ambulanssi ei tullut apuun |  | 0 | 0 |
| Avuton olo kun ei osaa auttaa |  | 0 | 0 |
| Huoli altistamisesta |  | 0 | 0 |
| Huoli omasta terveydestä |  | 0 | 0 |
| Syyllisyyttä tartuttamisesat ja kuoleman tuottamisesta |  | 0 | 0 |
| Whats app ryhmän avulla tiesin arvasin että minulla on korona |  | 1 | 2 |
| HAASTATTELU23 |  | 0 | 0 |
| Asioista kerrottiin jos tarvetta oli eli mitenkään yleisesti |  | 1 | 1 |
| En ajatelluit heti koronaa koska ajattelin että olis pitänyt olla ulkomailla |  | 1 | 1 |
| En saanut tietoa miten minun olisi oireettomana tullut toimia |  | 1 | 1 |
| Epäselvyyttä että missä voi olla ja mitä voi tehdä |  | 1 | 3 |
| FB postaus kaiken jälkeen |  | 1 | 1 |
| Henkinen puoli että mitä uskaltaa tehdä |  | 0 | 0 |
| huoli itsestä |  | 0 | 0 |
| Jatkuvaa oman tilan analyyisä kun varsinaista tietoa ei ollut |  | 0 | 0 |
| Kontakteille kerrottiin |  | 1 | 1 |
| Median seuraaminen auttoi että ei tullut yllättyksenä |  | 1 | 1 |
| Miettii että miten asian esittää että ei tule paniikkia |  | 0 | 0 |
| Olin lukenut paljon eli ei tullut yllätyksenä |  | 1 | 1 |
| Pelko altistamisesta |  | 1 | 1 |
| Pidensin karanteenia omaehtoisesti |  | 0 | 0 |
| Sosiaaliset verkot pienet ja liittyvät koronaan joten kaikille on kerrottu |  | 1 | 1 |
| Tieto koronasta myähään vaikka epäilystä oli |  | 0 | 0 |
| Whats app group auttoi keksimään miten voisi päästä testeihin |  | 1 | 1 |
| Whats app kautta tietoa ja mielenrauhaa että korona ei ole vaaralline lapsille |  | 1 | 2 |
| HAASTATTELU24 |  | 0 | 0 |
| Asumijärjestelyjä ei koska oltiin jo asuttu samoissa tiloissa yli viikko |  | 1 | 1 |
| Asumisjärjeestelyt vaikeita pienten lasten kanssa |  | 1 | 1 |
| Ei huolta koska oireet olivat mietoja ja tiedettiin että lapset eivät sairastu pahasti |  | 1 | 1 |
| Energiaa purettiin riitelemiseen lapset |  | 1 | 2 |
| Epäselvää karanteenin pituus |  | 0 | 0 |
| Epävarmaa kun ei tiedä onko sairastanut |  | 1 | 1 |
| Epävarmaa miten pitkän immuniteetin saa |  | 0 | 0 |
| Epävarmuus kun ei päästy testeihin |  | 0 | 0 |
| Epävarmuutta olenko sairastanut koronan |  | 1 | 1 |
| Halu informoida |  | 1 | 1 |
| Huoli ei voinut tavata isovanhempia |  | 0 | 0 |
| Huoli isovanhemmista joita ei voi nähdä |  | 1 | 1 |
| Huolissaan olenko latistanut koulussa yli 500ihmistä |  | 0 | 0 |
| Ihmiset ovat vaikeina eivät tiedä mitä sanoa |  | 1 | 1 |
| Kaverit on olleet varovaisia ja kyselleet vähän |  | 1 | 1 |
| Kerroin kontaktille joka oli shokissa ei esim kysellyt meistä mitään |  | 1 | 1 |
| Kerroin vapaasti kaikille kun asia varmistui kuten fudisjoukkueen vanhemille |  | 1 | 1 |
| kuorolaisilla oli niin epäilys oli |  | 1 | 1 |
| Logsitiikkaa ruoan suhteen hankalaa |  | 1 | 1 |
| Mediaa jaksaa seurata vain valikoiden |  | 1 | 1 |
| Meillä on nyt helpompaa kuin monilla muilla perheillä jotka ovat sairastaneet |  | 1 | 1 |
| Naapurit tarkisti oliko ok ettälapset tapas |  | 1 | 1 |
| Oireettomien tartuttaminen on vaarallista |  | 1 | 1 |
| Oli vain epäily koronasta Vaikeaa selittää lapsille miksie he eivät voi olla kavereitten kanssa |  | 1 | 1 |
| Olin seurannut koronaa ja tilanne huolestutti |  | 0 | 0 |
| Omatoimine karanteeni |  | 1 | 1 |
| Päätin että pakko tästä on uskaltaa liikkua |  | 1 | 1 |
| Ristiriitaiaisa ohjeita lasten lääkäri ja aikuisten lääkäri |  | 1 | 1 |
| Sairausasiat on yksityisasioita |  | 1 | 1 |
| Syyttelyä miksi et ole kertonut asiasta laajemmin kopu |  | 1 | 1 |
| Tiukkaa menevälle perheelle |  | 1 | 2 |
| Tunsi itsensä tappavaksi aseeksi |  | 1 | 1 |
| Töissä on tullut olo että ihmiset ajattelee että mitä tuokin on jo tullut töihin |  | 1 | 1 |
| Ulkoileminen Ei käyty missään edes rappukäytävässä |  | 1 | 1 |
| Vain lähimmille kolleegoille mutta ei oppilaiden vanhemmille |  | 1 | 1 |
| Vertaistukea lapset sairastaa ja vauvat sairastaa |  | 1 | 1 |
| Whats App oli todella tärkeä tuki |  | 1 | 1 |
| Ystävät pitävät etäisyyttä pitkään karanteenin jälkeen |  | 0 | 0 |
| HAASTATTELU27 |  | 0 | 0 |
| 27 ihmiset on peloissana kun kertoo että on sairatsnut koronan |  | 1 | 1 |
| 27 ihmiset pelkää koska eivät ole varmoja immuniteetista. sähän voit veiläkin tartuttaa |  | 1 | 1 |
| 27 ihmiset puuttuvat asioihin jotka eivät ole heidän tontin asioita |  | 1 | 1 |
| 27 kaikkein raskainta oireiden jälkeen oli pelko että tartuttaa |  | 1 | 1 |
| 27 kaverin sairaalameno herätti ajattelemaan että tämä voi olla jotain vakavaa |  | 1 | 1 |
| 27 kaverit pelkää nähdä meitä mutta ovat tapailleet omia riskiryhmävanhempiaan koko kevään |  | 1 | 1 |
| 27 poikkeuslaki julitus oli juhhlavaa ja tramaattista |  | 1 | 1 |
| 27 pystyin kertomaan enenmmän koska en ollut omalla käytökselläni tartuttanut muita |  | 1 | 1 |
| 27 päivikodin opettaja kavahti |  | 1 | 1 |
| 27 tauti vaan jatkuu ja jatkuu kun on huoli je epävarmuuss että koska se loppuu |  | 1 | 1 |
| 27 testin kautta saaa varmuuttaa että ei ole enää tartuttava |  | 1 | 1 |
| 27 tois paljon vaikutusta elämään jos tietäisin että en tartuta |  | 1 | 1 |
| 27 työkaverit olivat uteliaita |  | 1 | 1 |
| 27 vaikeinta että elettiin vaimon kanssa eri kuplissa |  | 1 | 1 |
| 27 whats app ja uutisfriikki |  | 1 | 1 |
| 27 vietettiin ykis ylimääräinen karanteeniviikko |  | 1 | 1 |
| 27 ystävän joutuminen sairaalaan pelotti |  | 1 | 1 |
| HIUS puhelinsoitto toi tirvaa tietoa sain Whats App ryhmältä |  | 1 | 1 |
| Testeihin kulkeminen julkisilla |  | 1 | 1 |
| Whats app poisti epävarmutta |  | 1 | 1 |
| HUOLI OMASTA TILASTA |  | 0 | 0 |
| En ollut itsestäni huolissani koska en ollut riskiryhmässä |  | 1 | 1 |
| Huoli kulmi sen mukaan mitä oireita oli |  | 1 | 1 |
| Huolta että jos tauti on tehnyt pysyviä muutoksia elimistöön |  | 1 | 1 |
| Todettiin että pitää mennä testiin koska on ollut epidemiaalueella joten valehtelin oireet |  | 1 | 1 |
| Voiko itselle käydä huoosti huolen takia seurattiin mediaa |  | 1 | 1 |
| IHMISTEN PELKO |  | 0 | 0 |
| Aistii että ihmiset eivät halua olla lähellä |  | 1 | 1 |
| Ei olla huomattu mutta ei olla ketään tavattukaan |  | 1 | 1 |
| En tiedä miten paljon leimaa on mutta uskoisin että jos ihmiset tietävät niin pelästyisivät enemmänkin |  | 1 | 1 |
| Ihmiset on varovaisia |  | 1 | 1 |
| Ihmisillä tulee primitiivisä rekatioita kun he kohtaavat pelkoa |  | 1 | 1 |
| Jotku ystävät eivät halua tavata |  | 1 | 1 |
| Jotkut vanhemmista oli hysteerisiä ja halusi tarkkoja ohjeita ja moneen kertaan piti sano että ei ole sellaisia ohjeita saatu |  | 1 | 1 |
| Jätetään tavarat parin metrin päähän ja äkkiä kipitetään kotiin |  | 1 | 1 |
| Kaverit pelkäs isän puolesta mutta ei mitään isoja reaktioita |  | 1 | 1 |
| Kukaan ei oo halunnut tulla lähellekään karanteenin aikana |  | 1 | 1 |
| Muutamien kanssa piti kesksuetlla mahdollisesta tartunnasta mutta tapaamisesta oli ollut kauan L |  | 1 | 1 |
| Taloyhtiölle selvisi jolloin alkoi portaikkojen desifiointi |  | 1 | 2 |
| Tartunna jälkeen koko kerros tyhjennettiin ja kaikki kottin minun tartunnan takia |  | 1 | 1 |
| Yliampuvaa reagointia |  | 1 | 1 |
| IHMISTEN VIHA |  | 0 | 0 |
| vihantunteita ja syyttelyä olisin voinut tuntea jos vauvan tila olisi ollut todella vakava |  | 1 | 1 |
| Yksityisyys piti suojata vihalta |  | 1 | 1 |
| KORONAN JÄLKEEN |  | 0 | 0 |
| Aluksi jänniitti että mitä tapahtuu kun tartuntoja oli niin vähän |  | 1 | 1 |
| Ei suuria muutoksia koronan jälkeen ehkä vähän enemmän varovaisuutta L |  | 1 | 1 |
| Fyysinen kunto on edelleen heikko |  | 1 | 2 |
| Korona on tuonut uusia ystäviä kuten futisjengi |  | 1 | 1 |
| Koulut ja sosiaaliset ongelmat huolestuttaa |  | 1 | 1 |
| Kukaan ei sanonut että jos hajuaistia ei kehotä se voi jäädä niin |  | 1 | 1 |
| Kukaan ei sanonut silloin että kannattaa oleskela eri huoneessa kuin muuta prehenejäsenet |  | 1 | 1 |
| Mikään ei tule muutumaan elämässämme |  | 1 | 1 |
| Muuttunut arvostan nyt sosiaalisia kontakteja enemmän kuin koskaan |  | 1 | 1 |
| Olen ollut ihan normaalisti kavereitten kanssa karanteenin jälkeen |  | 0 | 0 |
| Oma tilanne ei normalisoidu ennen kuin koko maan tilanne on normalisoitunut |  | 1 | 1 |
| Ottaa asian vakavammin tulevaisuudessa |  | 1 | 1 |
| Pesen käsiä enemmän (lapsi) |  | 1 | 1 |
| Pieniä koskeutsu muutoksia voi tulla mutta kyllä elämä lähtee rullaamaan niin kuin ennen |  | 0 | 0 |
| Sairaus jatkuu edelleen jossain määrin |  | 1 | 1 |
| Taloudellisiin ongelmiin ei puututa tarpeeksi |  | 2 | 2 |
| Tarvitaan kokonaisvaltaisen hoidon suunitelma ei vain virushoito |  | 1 | 1 |
| Taudin käyneenä on jonkinlainen helpottunut olo |  | 1 | 2 |
| Tulevaisuudessa ei suuria muutoksia ehkä enemmän käsihygieniaa ja vähemmän kättelyitä |  | 1 | 1 |
| Tulevaisuudessa ei tilaa matkoja yhtä helposti ja ajattelee käsienpesua enemmän |  | 1 | 1 |
| Tulevaisuudessa kiinnittää enenmmän huomiota maailmalta kantautuviin uutisiin |  | 1 | 1 |
| Tulevaisuudessa miettii joukkokohtaamisia ja seuraa viranomaisten ohjeita |  | 1 | 1 |
| Tulevaisuudessa vähemmän lentämistä |  | 1 | 1 |
| Tulevaiuudessa tulee lama ja se voi synnyttää sodam |  | 1 | 1 |
| Vaikutukset yhteiskuntaan |  | 0 | 0 |
| Yhteiskunnallinen |  | 1 | 2 |
| Yhteiskunnallinen huoli eteenkin lapsista on suuri |  | 1 | 1 |
| KUOLEMANPELKO |  | 0 | 0 |
| Ei pelottanut kun ei ole perussairautta |  | 1 | 1 |
| Ei pelottanut missään vaiheessa |  | 1 | 1 |
| Huolestunut vauvan takia |  | 1 | 1 |
| Huolestutti kun tauti vain paheni mutta testihinkään ei päässyt |  | 1 | 1 |
| Korona pelotti siinä vaiheessa kun saattiin tietää (lapsi) |  | 1 | 1 |
| Kuolema kävi mielessä mutta kun äiti on kuitenkin perusterve L |  | 1 | 1 |
| Kuolema tuli mieleen tv uutisvirrasta Espanjan ja Italian tilanteesta |  | 1 | 1 |
| lapset pelkäsi aluksi |  | 1 | 2 |
| Olin peloissani silloin kun molemmilla vanhemilla oli kuumetta |  | 1 | 1 |
| Pelkäsin että kun tämä on uusi viirus että sillä ei olis vaikutusta vauvan kehitykseen |  | 1 | 2 |
| Pelkäsin kuolemaa kun tauti oli pitkä eivätkä oireet hellittäneet |  | 1 | 1 |
| Pelotti että mitä jos hengitys menee vielä pykälän huonommaksi |  | 1 | 1 |
| Pelotti hengeahdistus |  | 1 | 1 |
| Vaimon kovat oireet alkoivat pelottaa |  | 1 | 1 |
| LAPSET |  | 0 | 0 |
| Ajattelin että tämä on niin kuin normaali flunssa |  | 1 | 1 |
| Ei ole epämiellyttävää mennä ulos |  | 1 | 1 |
| Eka kerta kavereita vähän jännitti tavata |  | 0 | 0 |
| En ole kertonuty kaikille koska en halua tästä kohua |  | 1 | 1 |
| Epävarmuuttaa kun tidetään koronasta niin vähän |  | 1 | 1 |
| Haluais että koulu alkais pian ja näkis kavereita |  | 1 | 1 |
| Hankalinta oli olla kotona ja vähän tekemistä |  | 1 | 1 |
| Huojentavaa että karanteeni päättyi |  | 0 | 0 |
| Huoli jos olen itse tartuttanut korona muihin |  | 1 | 2 |
| Hyvä tunne että me ollaan sairastettu |  | 1 | 1 |
| Juteltiin kavereitten kanssa keskenään ei valmenjan kanssa |  | 1 | 1 |
| Jännitti jos me ollaan saatu tartunta kun se oli ihan alkuaikoja |  | 1 | 1 |
| Karanteeni jälkeen en ajatelut että tartuttaisin vaikka en voi tietää kun oireita ei koskaan olut |  | 1 | 1 |
| kavereitten kanssa keskustelttiiin miten paljon sairatuu ja mitä jos koulut menee kiinni |  | 1 | 1 |
| kaverit oli pikkasen peloissaan alussa |  | 1 | 1 |
| Oli ihan ok että äiti kertoo koronasta koulussa |  | 1 | 1 |
| Kaverit otti etäisyytt ei halunnu olla tekemisissä |  | 1 | 1 |
| keskustelu HJK kavereitten kanssa ei ole jatkunut |  | 1 | 1 |
| Koulujen bussimatkat huolestuttaa |  | 1 | 1 |
| Luokan kanssa on juteltu ehkä jopa enemmän kuin tavallisesti |  | 1 | 1 |
| Mietittiin että jos tartunta tuli HJK pojan tartunnan vuoksi |  | 1 | 1 |
| Mietittiin mitä yhtyksiä meidän joukkuuella ja toisella joukkueella oli |  | 1 | 2 |
| oli mulle kerroottu että se on vaarallinen ja jossain päin maailmaa mutta en mä olis ajatellut että se tulis meille |  | 1 | 1 |
| olin ihmeissäni että miksi meillä olis korona |  | 1 | 1 |
| On ollut kiva että on ollut aikaa olla kotona |  | 1 | 1 |
| Pelkona että jos joku läheinen kuolee |  | 1 | 1 |
| Tartunta tuli todennäköisemmin konsertista |  | 1 | 1 |
| Testitulosten jälkeen voin kertoa enemmän ihmisille |  | 0 | 0 |
| Tylsää ei saanut tavata ketään, poistoa kotoa tai käydä kaupassa |  | 1 | 1 |
| Vanhemmat otti selville koronasta ja kertoi lapsille |  | 1 | 1 |
| viestintä Whats Appin ja snapchatin kautta |  | 1 | 1 |
| NEGATIIVISET REAKTIOT |  | 0 | 0 |
| Aluksi tulee olemaan leimaa joka sitten karisee pois myöhemmin |  | 1 | 1 |
| Ihmiset on niin tunteellisia lasten kanssa |  | 1 | 1 |
| Ihmiset seisoivat kaukana meistä |  | 1 | 1 |
| Ihmiset vahtii |  | 1 | 3 |
| Jos koulussa tai fudis joukkueessa joku olisi kuollut niin asia olisi eri |  | 1 | 1 |
| Nuorille karanteeni on ollut turhauttavaa |  | 1 | 1 |
| Opin tietämään ketkä ovat todelliset ystävät |  | 1 | 1 |
| Sana kiersi työpaikalla |  | 1 | 2 |
| Stigma oma veli ei menny ostamaan hänelle ruokaa |  | 1 | 1 |
| Työkaverit pelkäs |  | 1 | 1 |
| OMA AVOIMMUUS PALKITTIIN |  | 0 | 0 |
| Ei mahdolliselle tartuttamiselle voi mitään |  | 1 | 1 |
| En halua saada leimaa otsaan että tiedettiin mutta ei tehty mitään asialle |  | 1 | 1 |
| En tartuttanut ketään työpaikalla |  | 1 | 1 |
| Ihmiset ajattelee että jos pysytään kotona niin sitten tämä on ohi |  | 1 | 1 |
| Jos oltais odotettu viranomaisten opastusta niin siinä olisi voinut sattua pahempikin |  | 1 | 1 |
| Jotku vanhemmat oli poliiseja vaikka me ilmoitettiin heti alussa |  | 1 | 1 |
| Jäätiin omaehtoiseen karanteeniin |  | 1 | 1 |
| Kansalaisvelvollisuus kertoa kaikille |  | 1 | 1 |
| Kerroin kaikille joita olin tavannut |  | 1 | 2 |
| Kerroin kaikille koska tämä on globaali pandemia joka vaikuttaa yhteiskunnallisesti |  | 1 | 1 |
| koska kerroimme heti niin saimme paljon kannustusta |  | 1 | 1 |
| Lähdin töistä heti sanomatta mitään kenellekään |  | 1 | 1 |
| Meidän täytyy kantaa vastuu ja tehdä pisin mahdollinen karanteeni |  | 1 | 1 |
| Mun velvollisuus on kertoa tartuntaketjuista |  | 1 | 1 |
| Olen pitänyt huolemn sitä että emme tee asioita joista meitä voisi syylistää |  | 1 | 1 |
| Pitää ilmoittaa koska tämä on pandemia |  | 1 | 1 |
| Pitää olla kotona eikä saa poistua |  | 1 | 1 |
| Päätettiin itse kaveriporukalla että ei mennä minnekään |  | 1 | 1 |
| Suomalaiset on kuuliaisia |  | 1 | 1 |
| Tarttuja hoiti asian hyvin |  | 1 | 1 |
| Tartuttaja puski itsensä testeihin |  | 1 | 1 |
| Velvollisuus käydä testeissä vaikk en uskonut että on korona |  | 1 | 1 |
| Viestittelimme kaikille asiasta vaikka sanottiin että ei tarvitse |  | 1 | 1 |
| PAHINTA |  | 0 | 0 |
| Makuaistin meno oli melkein suurin paha koko hommassa |  | 1 | 1 |
| PELKO KERTOA KORONASTA |  | 0 | 0 |
| Ei kerrottu koronasta somessa |  | 1 | 1 |
| Ei olla kerrottu naapureille kun ei olla oltu kekenkään kanssa tekemisissä |  | 1 | 2 |
| En ole laittanut tietoa koronasta FB tai Twitteriin vain Whats App löhimpien ystävien kanssa |  | 1 | 1 |
| Ilmoitettiin positiivisesta testitä heti kaikille häävierialle |  | 1 | 1 |
| Ilmoitin itse synttärijuhlavieraille asiasta |  | 1 | 1 |
| Ilmoitin toissä kaikiille jolloin kaikki jäivät kotiin |  | 1 | 1 |
| Isä lähetti vietsin kavereille mutta ei ne oo paljoa kyselleet |  | 1 | 1 |
| Itse pelkää että katsookohan nuo naapurit nyt |  | 1 | 1 |
| Kerroin testitulosta odottaessani että mulla saattaa olla koronaa |  | 1 | 1 |
| Kerroin työkavereille että eivät ihmettele miksi olen posi kuukauden |  | 1 | 1 |
| Kerrottiin koronasta jos joku kysy |  | 1 | 1 |
| kerrottin nappuruston Whats App ryhmässä että meillä on korona ja viemme roskat hanskat kädessä ja pestään käsiä |  | 1 | 1 |
| Kertoivat naapureille koska tarvitsivat apua auton parkkeeramisessa |  | 1 | 1 |
| Korona statuksesta ilmoitettiin what app ryhmälle heti tulosten saatua |  | 0 | 0 |
| Kynnys on edelleen eli katson kenenlle asiasta kerron |  | 1 | 1 |
| Lapsi pelkäsi mennä kouluun |  | 1 | 1 |
| Leima pelkotila |  | 1 | 1 |
| Me haluttiin ottaa aktiivinen rooli kun meillä on yhteiskunnassa monia eri rooleja |  | 1 | 1 |
| Mies ehdotti että ei kerr Hr enne kuin on varmaa että ei lietso paniikkia mutta kerroin |  | 1 | 2 |
| Mietin että aletaanko meitä nyt karttamaan |  | 1 | 1 |
| Mietin että minkähänlaisia kommennteja tulee |  | 1 | 2 |
| Mä kerroin heti äiti lapsi kaverille että tämmönen on käynyt |  | 1 | 1 |
| Noloa soittaa HR päällikölle |  | 1 | 1 |
| Olen kertonut asiasta en julistanut varsinaisesti |  | 1 | 1 |
| Olen miettinyt kaikkien kohdalla kannattaako kertoa mutta olen päätynyt siihen että kannattaa |  | 1 | 1 |
| Olin itse yhtedessä kontakteihin koska HUSin soitto tuli myöhässä |  | 1 | 1 |
| Ollaan itse sanottu että meidän lasten kanssa ei voi olla |  | 1 | 1 |
| Pyysin että naapurit eivät paljastaisi vähän kaukaisemmille taloyhtiöille |  | 1 | 1 |
| Päätin että syyllisyydentunne pois . sille ei mitään voi lopetin saomasta sorru |  | 1 | 3 |
| Suositeltiin että muille ei kerrota kuin niille joille on pakko |  | 1 | 2 |
| TIETO PERHEEN KORONASTA LEVISI NOPEASTI |  | 0 | 0 |
| Tieto meidän koronasta levisi todella nopeasti meidän asunaluella |  | 1 | 1 |
| Voi tulla koronaopettaja leima mutta en usko että se tulee olemaan kauhean negatiivinen |  | 1 | 1 |
| Ystäville, ristiäivieraille ja papille kerrottiin |  | 1 | 1 |
| PELKO TAVATA MUITA KARANTEENIN JÄLKEEN |  | 0 | 0 |
| Ahtaat rappukäytävät niin ei sitten vittinyt |  | 1 | 1 |
| Edelleen pidetään välimatkaa vanhempaan äitiin |  | 1 | 1 |
| En halua tavata äitiä enne kuin jälkikaranteeni on ohi |  | 1 | 1 |
| Epäselvää että kuinka kauan me voidaan tartuttaa |  | 1 | 1 |
| Ikävintä on se että vieläkään ei olla voitu käydä siovanhempien luona |  | 1 | 1 |
| Itsenäisesti nankkeuduin kokeisiin ja jäin ersityksiin odottamaan tuloksia |  | 1 | 1 |
| Juoksin nopeasti käytäväss että en tapaa ketään tai tartuta ketään |  | 1 | 1 |
| Jännitti käydä kaupassa karanteenin jälkeen |  | 1 | 1 |
| Jännittää halata |  | 1 | 2 |
| Jää varautuneisuus että jos tauti kuitenkin tarttuu |  | 1 | 1 |
| Karanteenin jälkeen eka viikolla ei ollut sellaista oloa että olisin halunnut mennä kauppaan kövin vain kävelemäss oli nuhaa |  | 1 | 1 |
| Kierrän ihmisiä kauempaa |  | 1 | 1 |
| Kiersin ihmiset todella kaukaa |  | 1 | 1 |
| Kun olen nähnyt karanteenin jälkeen ihmisiä olen sanonut että voin vielä tartuttaa |  | 1 | 1 |
| Mietin voinko tavata äitiä |  | 1 | 1 |
| Mietitään että voiko korona elää vaatteissa tai jotain kun mies tulee viikon perässä |  | 1 | 1 |
| Nähnyt isää välimatkan päästä |  | 1 | 1 |
| Oltiin varovoaisia koska on niin ristiriitaisia tietoja tartuttavuudesta |  | 1 | 1 |
| Omissa oloissa koska ei ole tietoa kaunako se voi tarttua |  | 1 | 1 |
| Onneksi ei menty töihin |  | 1 | 1 |
| Pelkää tartuuvansa kun menee ulos ja näkee lapsia |  | 1 | 1 |
| Pelkään että tulee tutkimustuloksia että voinkin tartuttaa |  | 1 | 2 |
| Pelottaa että jos on joku mahdollisuuss vielä tartuttaa |  | 0 | 0 |
| Pelotti että saan itse oireita L |  | 1 | 1 |
| Pelotti tavata ihmisiä karanteenin jälkeen |  | 1 | 1 |
| Tavannut vain tartunnan saanutta |  | 1 | 2 |
| Tekee valintoja kun menee ulos |  | 1 | 1 |
| Tunne olla kauempana on molemminpuolinen |  | 1 | 1 |
| Turvallista tavata ihminen joka on taudin sairastanut ja samasta lähteestä |  | 1 | 2 |
| Vain miehen kanssa liikutaan vaikka testien mukaan en ole sairas |  | 1 | 1 |
| Yritti olla koskettamatta mihinkään tai hengittämättä kun vei roskia |  | 1 | 1 |
| POSITIIVISTA |  | 0 | 0 |
| Ei pelottanut koska ei ole riskiryhmässä |  | 1 | 1 |
| Hyvä sairastaa nyt kun hoitokapasiteettiä on |  | 1 | 2 |
| Karanteeniaka oli ihana rauhoittavaa |  | 1 | 1 |
| Karanteenin aikana lähennyttiin |  | 1 | 1 |
| Karanteenissa nautin siitä että aikaa oli äärettömästi |  | 1 | 1 |
| KORONA EXPERTTI |  | 0 | 0 |
| korona ammattilainen |  | 1 | 1 |
| Koronatestin jälkeen pystyn informoimaan kaikkia kavereiat että mulla ei oo L |  | 1 | 2 |
| Me ollaan nyt kaikkein turvallisinta seuraa mitä löytyy |  | 1 | 1 |
| Positiivista että on saanut rauhoittua raskausaikana |  | 1 | 2 |
| Työmarkkinoilla vahvoilla kkun on immuuni |  | 1 | 1 |
| POSITIIVISTA KANNUSTUSTA |  | 0 | 0 |
| Fudis vanhempien ryhmä räjähti viesteistä paljon positiivsta |  | 1 | 1 |
| REAKTIOT KORONAAN |  | 0 | 0 |
| Ajateltiin että kun ei olla oltu epidemiaalueella niin se ei voi olla koronaa |  | 1 | 1 |
| AJATTELIN HETI KORONAA |  | 0 | 0 |
| Ajattelin koronaa kun ensimmäinen italian korona cae ilmoitettiin |  | 1 | 1 |
| Median raportoinni jälkeen ajatelin asiaa |  | 1 | 1 |
| Ajattelin alussa että tämä on ihan perusflunssa vain |  | 2 | 2 |
| Ajattelin ennen koronaa että pahimmillaan kuin influenssaa |  | 1 | 1 |
| Ajattelin että hyvä että saatiin se nyt |  | 1 | 1 |
| Ajattelin että korona on niin kuin influenssa |  | 1 | 1 |
| Ajattelin että kuvittelin koronan oireet kun olin lukenut siitä niin paljon |  | 1 | 1 |
| Ajattelin heti että se o koronaa kun faisa sai kuumeen kun siitä puhuttin niin paljon telkkarissa L |  | 0 | 0 |
| Aluksi ajattelin että flunssaa mutta koska jo niin paljon kesksuteltiin niin en voinut olla ajattelematta koronaa |  | 1 | 1 |
| Alussa ajattelin että on koronaa ja sitten Italian kuvat pysäytti että jos se onkin jotain vakavampaa |  | 1 | 1 |
| ei ollut yllätys |  | 1 | 1 |
| ei osattu ajatella voisko meillä olla korona |  | 1 | 2 |
| Ei tiedetty paljoakaan koronasta joten en osananut pelätä mitenkään miehen puolesta |  | 1 | 1 |
| En ajatellut että korona voisi tulla omalle kohdalle L |  | 1 | 2 |
| En ajatellut että tulisi omalle kohdalle kun ajattelee olevansa haavoittumaton |  | 1 | 3 |
| Eätodellinen niin kauan kuin tulos tuli koska Suomessa oli niin vähän tartuntoja |  | 1 | 1 |
| Hallusinaatioita |  | 1 | 1 |
| harvalle ei riskiryhmäläiselle vakava |  | 1 | 1 |
| Heitin läppää työmatkalla Virossa että onkohan koronaa |  | 1 | 1 |
| helpotus tietää vastaaineista |  | 1 | 2 |
| Heti ensioireiden jälkeen ajatteli koronaa |  | 1 | 1 |
| Ihmisillä on eri reaktoitua jotku pelkää ja jotkut ei |  | 1 | 1 |
| Jotku kaverit ottaa pandemian vakavammin eli ne on kotona ja toiset tapailee ystäviä ja tosi vähän käytetää jukisia N |  | 1 | 1 |
| Jännää että tämä on maailmalaajuinen josta kaikki yrittää selvitä |  | 1 | 1 |
| Kaverit joita ei ole testattu on varmoja että he eivät voi saada sitä |  | 1 | 1 |
| Kaverit sanoo että saataispa mekin niin pääsis siitä |  | 1 | 1 |
| Kaverit toivoo että ne olis sairastaneet tämän |  | 1 | 1 |
| Konsertin aikana vitsailtiin että nythän ei saa kätellä mutta todellisuudessa kaikki pöräs aulassa ihan normaaliin tapaan |  | 1 | 1 |
| Korona harmittaa L |  | 1 | 1 |
| koskaan ei tiedä |  | 1 | 1 |
| Kun kaverit kuuli niin ne oli neutraaleja L |  | 1 | 1 |
| Kun kuulin että kolleega työpaikalla oli sairas tajusin että myös minulla on korona |  | 1 | 1 |
| Kun kuulin tartunnasta niin ajattelin että tämä on yksi influenssa vaan |  | 1 | 1 |
| Kun oli sairas niin ihmetteli hallituksen hitaita reaktioita |  | 1 | 3 |
| Masennus osa taudinkuvaa |  | 1 | 1 |
| me suhtaudutaan siten että meillä on vasta aineita ja voidaan nähdä kavereita |  | 1 | 1 |
| Mustaa huumoria |  | 1 | 1 |
| Nuoret pelkää että koko kesä perutaan |  | 1 | 1 |
| Nuorille eristäytyminen on rankaa mutta ei niitä pelota L |  | 1 | 1 |
| Oli helpotus saada vihoin tietää mikä vaivaa |  | 1 | 1 |
| Oli kiva tietä että mulla oli korona |  | 1 | 1 |
| Oli kiva tietää testitulos vaikka me oltiin oltu sairatiat jo puolitoista viikkoa |  | 1 | 2 |
| Olin ajatellut että saan tämön varmasti kun olen koulussa opettajana |  | 1 | 1 |
| Olin lukenut oireista todella paljon joten olin melko varma että minulla on korona |  | 1 | 1 |
| Oltiin hyvin tietoisia Kiinan tilanteesat eli asia ei tullut yllätyksenä ollenkaan |  | 1 | 1 |
| Oppilaita ei asia kauheasti kiinnosta |  | 1 | 1 |
| Parempi että kaikki vaan sarastaisi tämän taudin |  | 1 | 1 |
| Pidin epätodennäköisenä että mulla olis koronaa |  | 1 | 1 |
| Pidin varmana että mulla on korona |  | 1 | 1 |
| SHOKKI TAI PANIIKKI |  | 0 | 0 |
| Shokki ja paniikki että mitä nyt tapahtuu |  | 1 | 2 |
| Testitulos ei ollut yllätys koska hiihtoporukan Whats Appissa asiasta käytiin keskustelua |  | 1 | 2 |
| Työni puolesta joudin pohtimaan isoja kokonaisuuksia jonka vuoksi olin perheessä myös se joka regoi korona tilantesseen ensiksi ja otti lapset pois koulusta |  | 1 | 1 |
| USKO ETTÄ OIREET EI TARTU NUORIIN TAI LAPSIIN |  | 0 | 0 |
| Ei ole käsitys muuttunut sen piti olla vaarallinen riskryhmille N |  | 1 | 1 |
| En pelännyt vanhempien kohdalla kuolemaa kun eivät ole riskiryhmiä |  | 1 | 1 |
| Kaverit ei pelkää kun ne on kuulleet että oireet on vähäiset lapsilla L |  | 1 | 1 |
| Virus on älykäs |  | 1 | 1 |
| VITSAILTIIN |  | 0 | 0 |
| Vitsailtiin että joo varmaan tää on nyt sitten koronaa |  | 1 | 1 |
| Vähättelin koronaa enne kuin sen tuli omalle kohdalle |  | 1 | 1 |
| YLLÄTTYNYT KORONASTA |  | 0 | 0 |
| Olin yllättynyt että iskällä oli korona |  | 1 | 1 |
| RISKIKOMMUNIKAATION VAIKUTUKSET |  | 0 | 0 |
| En seuraillut mediaa paljoakaan |  | 1 | 1 |
| EN SEURANNUT MEDIAA |  | 0 | 0 |
| Suojaan itseno siten että en lukenut liikaa |  | 1 | 2 |
| En tiennyt aluksi paljon mitään L |  | 1 | 1 |
| Enemmän tietoa vaimon Whats App ryhmästä |  | 1 | 1 |
| Ennen tartuntaa matkustettiin paljon ja pohdittinko voidaako matkustaa ja saadaanko virus |  | 1 | 1 |
| Etsin tietoa paljon itse |  | 1 | 1 |
| halu seurata, ymmärtää ja jakaa tietoa |  | 1 | 1 |
| Halusin kertoa kun se tarttui niin helposti siellä häissä. halusin olla vastuuntuntoinen |  | 1 | 1 |
| Ilmoitin FBssä koronasta ja ihmiset on tosi kiinnostuneita |  | 1 | 1 |
| Informaatio oli joskus ristiriitaista |  | 1 | 1 |
| Jaoin pojille vaimon kautta saatavaa tietoa |  | 1 | 1 |
| Kaikki Whats App keskustelut muuttuivat Whats App keskusteluiksi |  | 1 | 1 |
| Katsottiin sairaina Italian tilannetta ja oltiin paniikissa |  | 1 | 1 |
| Kavereitten kanssa ollaan puhuttu kun se on niin pinnalla nyt N |  | 1 | 1 |
| kevyttä vireyttä ylläpitävää Whats App keskustelua miesten kanssa |  | 1 | 1 |
| Korona oli ollut mielessä ja asiasta oli käyty keskustelua jo helmikuussa |  | 1 | 1 |
| Korona uutisointi oli pelon lietsontaa |  | 1 | 1 |
| Koronasta kertominen sai aikaan hässäkän ja viron tehdas suljettiin |  | 1 | 3 |
| Koulussa kerrottiin koronsta siihen tylliin että ei kannata pelätä. se oli erilaista kun lastenuttiset |  | 1 | 1 |
| Käsitys taudinkuvsta muuttui Whats App kautta kun uusista tartunnoista keskusteltiin |  | 1 | 1 |
| Kävi mökillä kertomatta Whats App ryhmälle |  | 1 | 2 |
| Lasten Whats App ryhmissä oli kamalia huhuja koronasta |  | 1 | 2 |
| Luin ja katsoin tvtä paljon ennen ja koronan aikana |  | 1 | 1 |
| Media haastattelun jälkeen syyteltiin paljon häävierasta |  | 1 | 1 |
| Media ja regointi on tuonut paljon ristiriitaisia tunteita |  | 1 | 1 |
| Medialle haastatelu yhteiskunnallisesta perpektiivistä |  | 2 | 2 |
| MEDIAN KANSSA YHTEISTYÖ |  | 0 | 0 |
| Koulun karanteeni ja futisjoukkueelle ilmoittaminen sai aikaan lehdistön ajojahdin |  | 1 | 1 |
| Miesten Whats App yritti nähdä sen valoisan puolen |  | 1 | 2 |
| Naisten Whats Apissa keskusteltiin kaikkein kauheimmat scenariot |  | 1 | 1 |
| Oli lukenut oireista ja tiesi mitä odottaa |  | 1 | 1 |
| olin lukenut koronasta |  | 1 | 1 |
| Ollaan keskusteltu Whats Appissä vähän julkkisien koronasta L |  | 1 | 1 |
| Ollaan puhuttu medialle näyttääksemme että tämä leviää kauhean helposti |  | 1 | 1 |
| Ollaan vedetty yhtä köyttä ja annettu haastatteluja medialle |  | 1 | 1 |
| Positiivinen tulos ei tuntunut pelottavalta koska siitä oli jauhettu jo usemapi päivä |  | 1 | 2 |
| SEURASIMME KORONAA JO ENNEN OMA EPISODIA |  | 0 | 0 |
| Korona tieto mediasta ja tieteellisä julkaisuja luin paljon koska siskoni ovat mikrobiologeja. se vaikuutti keskittymiskykyyn |  | 1 | 1 |
| Luin ulkomaalaisista lehdistä tapaukista halusin tietää kestosta oli vähän tietoa |  | 1 | 1 |
| Tiedettiin Kiinan tilanteesta seurattiin kv uutisia niin kuin niitä seurataan ja ystäväperhe astman takia todella valveutunut asiasta |  | 1 | 1 |
| Seurattiin media ja jaettiin valtavasti tietoa Whats Apissä |  | 1 | 1 |
| Tartunnan jälkeen akoi keräämään tietoa |  | 1 | 1 |
| Tartuttaja oli valveutunut |  | 1 | 2 |
| testaus tilanteessa keskustelin |  | 1 | 1 |
| Tieto televisiosta jossa puhutttiin oireista L |  | 1 | 1 |
| Uutisten negatiiviset uutiset voivat masentaa |  | 1 | 1 |
| vaikeinta ennen testituloksia selittää lapsille miksi he eivät voi tavata ystäviä |  | 1 | 1 |
| valtava googlatus ja What App ryhmään linkataan kaikki |  | 1 | 1 |
| Whats App group vertaistukea kun ryhmässä oli kolme muuta sairastunutta vauvaa |  | 1 | 1 |
| Whats App keskustelua enennmmän kuin tavallisesti |  | 1 | 1 |
| Whats App kommunikointi |  | 1 | 3 |
| Whats App oli hyvää vertaitukea ja vitsejä väännettiin |  | 1 | 1 |
| Whats App ryhmissä tulevaisuuden spekulointeja |  | 1 | 1 |
| Whats app ryhmiä oli erilaisia |  | 1 | 1 |
| Whats App ryhmä joka oli kv ja tehtiin kv vertailua |  | 1 | 2 |
| Whats App ryhmän kautta sai parhaat tiedot |  | 1 | 1 |
| Whats App ryhmässä jaettiin tietoa eli tiesin tosi paljon heti aluksi |  | 0 | 0 |
| Whats App ryhmässä oli kaikki ystävät ja tuttavat ja sieltä sai vertaistukea |  | 2 | 2 |
| Whats App ryhmässä oli keskustelua että kukaan ei tiedä miten kauan voi tartuttaa |  | 1 | 2 |
| Whats App ryhmäsä vertailittin oireita ja linkaattiin artikkeleja |  | 1 | 1 |
| Whats App verstaustuki |  | 1 | 1 |
| Whats APpin kautta kuuli vaarallisista oireista jotka sittne pelotti jos tulee miehelle |  | 1 | 1 |
| Viestiminen siitä että nyt sulla on lupa olisi tärkeää |  | 1 | 1 |
| Yhdessä on haluttuu muuttaa vääriä käsityksiä koronasta |  | 1 | 1 |
| Äiti levitti tietoa tästä |  | 1 | 2 |
| Äiti oli lukenut paljon ja kun oireet tulivat niin oli aika selvää että se on koronaa |  | 1 | 1 |
| Ärsyynnyin epidemian alussa liiasta uutisoinnista |  | 1 | 1 |
| SAIRAANHOITAJAN KOKEMUS |  | 0 | 0 |
| Coping- en ota yhteyttä ihmisiin |  | 1 | 2 |
| coping mechanims- rajotteita ei vielä silloin ollut |  | 1 | 1 |
| Coping mechanism että meillä on myös muuta lämää kuin sairaala |  | 1 | 1 |
| Ei eritysijärjetelyjä koska titeo tuli monta pöivää myöhemmin |  | 1 | 1 |
| ei huoleastuttanut erityisesti lasten tai perheen tilanne |  | 1 | 1 |
| Ei omilla somekanavilla piatsi toisen julkaisujen jakamista |  | 1 | 1 |
| En lopeta suukottelua ja halailua |  | 1 | 1 |
| En ole varma vasta-aineiden hyödystä joten en mene tapaamaan |  | 1 | 1 |
| En pitänyt tätä oikein minään |  | 1 | 1 |
| Epävarma olo kun piti palata töihin |  | 0 | 0 |
| Epävarmuuttaa paluusta töihin helpooti maskipakko |  | 1 | 1 |
| Huoli sairaasta ystävästä |  | 1 | 1 |
| Ihmiset pelkää koronan jälkeen |  | 1 | 2 |
| Ihmisten ensi rekatoiden jälkeen hakusi kertoa vielä vähemmän |  | 1 | 1 |
| isovanhempia ei olla nähty |  | 1 | 1 |
| Itsensä syyttelemistä |  | 1 | 1 |
| Itsnsä soimaaminen |  | 1 | 1 |
| Jos potilastartuntoja olisi tullut niin itssyytösten määrä olisi ollut valtava |  | 1 | 1 |
| Kaikki haluaa kuulla vasta-ainetestin tulokset |  | 0 | 0 |
| Kerroin lähimille tuttaville heti |  | 0 | 0 |
| Knnys kertoa kun ihmiset otti syyttely asenteen mutta myös uteliaisuus |  | 1 | 1 |
| Koronan jälkeen ei pelota koska ododtta että on immuniteeeti |  | 1 | 1 |
| Kynnys kertoa eirityisluokan lasten vanhemille |  | 1 | 2 |
| Lapsi oli etinyt kertoa jo kavereille jolloin laitoin perään viestiä |  | 1 | 1 |
| Oireeton lapsi pelkää että tartuttaa muita jos menee leikkimään |  | 1 | 1 |
| Pahoittaa mielensä ihmisten reaktoista |  | 1 | 2 |
| Pakko kertoa kontakteille |  | 1 | 1 |
| Pelko ja huoli että on tartuttanut potilaita |  | 1 | 1 |
| Pelkoa että ihmiset pitää huonona äitinä kun on laittanut lapset kouluun |  | 1 | 1 |
| Potilaiden pelko |  | 1 | 1 |
| Potilaiden puolelta syyttelyä |  | 1 | 1 |
| Puolustajiakin löytyi |  | 1 | 1 |
| Selitettiin kaverien vanhemmille että ollnaa oltu karanteenissa 3 viikooa eikö ole oireita |  | 1 | 1 |
| SOMEn kautta sai syytöksiä |  | 1 | 1 |
| Somen kautta tuli syyttelyä enennmään kuin työyhteisön sisältä |  | 0 | 0 |
| Syyttelyä muilta työpaikalla |  | 1 | 4 |
| Tiedon muuttuminen ei ole häiritsevää |  | 1 | 3 |
| Työpaikan oirettomat jotka eivät päässeet testeihin toivat jännitteitä työpaikalla |  | 1 | 2 |
| Uuteen rutiiniin adaptoitui |  | 1 | 1 |
| Whats App ryhmän vertaistuki |  | 1 | 2 |
| Whats App vertiastuli oli tärkeää |  | 1 | 1 |
| vihaista kesksutelua |  | 1 | 1 |
| Vähentänyt huoltalapsista kun näkee että ei ole lapsipotilaita |  | 1 | 1 |
| Yllätyksiä |  | 1 | 1 |
| Ei osannut ajatella ollenkaan taloudellisia vaikutuksia |  | 1 | 1 |
| Ei ollut ajatellut että se voisi tulla omalle kohdalle kun se oli Italiassa |  | 1 | 1 |
| Ei ollut mielessäkään että korona vois tulla häihin |  | 1 | 1 |
| ei tiedetty kuinka herkästi se tarttuu |  | 1 | 1 |
| En ajatellut että se voi nuorillekin olla noin rankka |  | 1 | 2 |
| Napakka päänsärky oli yllätys |  | 1 | 1 |
| Sairaalahoitoa tarvitsi moni ryhmästä |  | 1 | 1 |
| Vakava korona tarkoittaa hengitysvaikeuksia ja sairaalaa |  | 1 | 1 |
| Virheellinen käsitys että tämä on vaarallinen vain seniorikansalaisille |  | 1 | 3 |
| Yllättävää että oireet olivat epäselvät |  | 1 | 2 |
| Yllätys miten kovia oireet olivat |  | 1 | 1 |
| Yllätys että tartuu niin helposti tartutin kolleegan |  | 1 | 1 |
| Yllätys että tartuuja ei ollut ollut ulkomailla ja etttä se tarttui nopeasti |  | 1 | 1 |
| Yllätys että tervekin voi joutua teholle |  | 1 | 1 |
| Yllätys kun oma ystävä joutui hengityskoneeseen |  | 1 | 1 |
| SELVIYTYMISMEKANISMIT |  | 0 | 0 |
| Ajattelin että onko mitään järkeä senioreita suojella influnessan tapaiselta sairaudelta´90+´+09 890p+ |  | 1 | 1 |
| Epävarma olo että voinko mennä ja sitten ajattelin että mullahan oli lupa |  | 1 | 1 |
| Haluan korona todistuksen niin voi näyttää että mulla on vastataineet |  | 1 | 1 |
| Hökkätunnit |  | 1 | 1 |
| Kertomalla siirtää vastuun pois itseltään |  | 1 | 1 |
| Koko ajan tiesi muiden raskaana olevien voinnista |  | 1 | 3 |
| Kysyin luvan mennä ulos kun kaksi oireetonta päivää oli takana |  | 1 | 3 |
| kävelin hanskat kädessä ja huivi nenän edessä |  | 1 | 1 |
| Meillä on iso tukiverkko |  | 1 | 1 |
| Olen tavannut vain muita koronnaa sairastuneita aluksi |  | 1 | 1 |
| Oma työ oli ainoa pysyvä asia joka piti rutiinin koossa |  | 1 | 1 |
| Rannekkeet |  | 1 | 1 |
| Toimin sen hetkisen tiedon valossa joten en pidä toimintaani vastuuttomana |  | 1 | 1 |
| SOSIAALISET SUHTEET |  | 0 | 0 |
| Joka päivä soitettiin eli hyvissä käsissä |  | 1 | 1 |
| kavereitten kanssa on vietitelty päivittäin skyn kautta ja muuta L |  | 1 | 1 |
| Kommunikoiti ystävien ja siskojen kanssa on auttanut minua |  | 1 | 1 |
| Koulun rehtori teki määräyksen että teinit ei saa tavata joten teinejä ei näy missään |  | 1 | 1 |
| Miehellä ei ole suurta sosiaalista verkkoa sairaalssa hän oli samassa huonessa toisen koronapotilaan kanssa joka mahdolisiti vertaustuen |  | 1 | 2 |
| Muutenkin kavereita ethtii näkemään melko harvoin L |  | 1 | 1 |
| Onneksi meitä oli kaksi |  | 1 | 1 |
| Pallopelit katkolla harmittaa vaikka yhtedessä ollaan pelikavereihin |  | 1 | 1 |
| Tiivis ystäväverkko joka auttoi en ymmärrä miten ihmiset joilla sitä ei ole selviävät |  | 1 | 1 |
| Viestittely on mutenkin normaalia mutta varmaan palataan korona jälkeen siten että myös tavataan |  | 1 | 1 |
| Ystävät soitteli enemmän kuin normaalisti |  | 1 | 1 |
| STRESSI JA HUOLI MUISTA |  | 0 | 0 |
| Ei pelottanut korona raskaus vaan pelkäsin vanhempien ihmisten puolesta |  | 1 | 1 |
| En osannut pelätä meidän omasta puolesta kun oli huolissani vauvasta |  | 1 | 1 |
| Googlasin raivokkaasti raskaudesta |  | 1 | 4 |
| Huolestutti vähän että jos äiti joutuu sairaalaan |  | 1 | 1 |
| Huoli miten vanhukset jaksavat olla eristyksissä |  | 1 | 1 |
| Huoli nousi vanhemmista häävieraista |  | 1 | 1 |
| Huoli omasta miehestä helpottui kun hän pääsi sairaalaan. en olisi muuten jaksanut |  | 1 | 1 |
| Huoli riskiryhmälapsesta |  | 1 | 1 |
| Karanteeni ei ollut kauhean stressaavaa paitsipari viikkoa kun vanhemmat oli sairaina |  | 1 | 1 |
| Karanteeni oli helppoa etätyöaikaa paitsi huoli lapsen pitkäaikaisesta kuumeesta |  | 1 | 1 |
| Kiire ilmoittaa muille eli ei ehtinyt pelätä kun oli niin paljon käytännön hommaa |  | 1 | 1 |
| Kiire koko karanteenin ajan kommunikoida eri tahoille joka myös piti meidän perhesuhteet hyvinä |  | 1 | 1 |
| Lapsille on ollut kova paikka kun me molelmat olemme olleet sairaina kuumessa yhtäaikaa |  | 1 | 1 |
| Olin niin huolissani miehestäni ja arjen pyörttämisestä että en ehtinyt huolehtia itsestäni |  | 1 | 1 |
| Pelko mitä jos molemmat sairatuvat kuka huolehtii lapsista |  | 1 | 2 |
| Pelkäsin että oli tartuttanut vanhan äitini |  | 1 | 1 |
| Pelkää omien vanhemopien puolesta |  | 1 | 1 |
| Pelotti vähän vauvan puolesta kun testitulos oli positiivinen |  | 1 | 1 |
| Sressi ja huoli siihen asti kun potials pääsi |  | 0 | 0 |
| Stressi ja huoli isästä siihen asti kun hän pääs isairaalalan |  | 1 | 1 |
| Suurin huoli oli että kuka kuuluu riskiryhmään |  | 1 | 1 |
| vaikeinta kun puolison kunto romahti |  | 1 | 1 |
| Vaimo pelkäsi että jos se voi vaikuttaa vauvan kehitykseen |  | 1 | 2 |
| Vaimon raskaus tuotti pientä huolta |  | 1 | 1 |
| Vanhempien ihmisten vakavat sairastapaukset veivät paljon energiaa |  | 1 | 1 |
| SYYLLISYYS |  | 0 | 0 |
| Hyvä että taloyhtiölle kerrottiin koska aina pelotti kun vei roskia |  | 1 | 2 |
| Jos niistän niin ajattelen heti että eritänköhän jotain |  | 1 | 1 |
| Kolleega sai tarunnan jolloin tuli huoli että jos on ollut tartuttajana |  | 1 | 1 |
| Kun lähti ulos niin oli syyllinen olo |  | 1 | 1 |
| Menin töihin kun olin siirtänyt vastuun pomolle |  | 1 | 1 |
| Mietein olenko tartuttanut muita |  | 1 | 1 |
| Olemme tehneet oma aloitteisen jälkikaranteenin |  | 1 | 1 |
| Oli syyllinen olo kun lähti ulos ensimmäisen kerran |  | 0 | 0 |
| Olin huolissani että kenet olen tartuttanut |  | 1 | 1 |
| Pelkäsin että olen tartuttanut muita |  | 1 | 1 |
| PYYTELIN ANTEEKSI |  | 0 | 0 |
| Pyysin anteeksi altistetuilta |  | 1 | 1 |
| Vähitellen ymmärsin että tää tilanne on sellainen että ei voi mitään ja lopetin sori sanomiset |  | 0 | 0 |
| Syyllisyys ja huoli isästä joka oli saanut koronan piti kiireisenä |  | 1 | 3 |
| Työkaverit ei jää kotiin jos ei viranomainen opasta |  | 1 | 1 |
| Vaikutti siten että en saanut ensimmäistä ultrraa silloin kun olisi pitänyt saada |  | 1 | 1 |
| SYYT PANDEMIAAN |  | 0 | 0 |
| En usko että epidemia johtuu liikakuluktusesta |  | 1 | 1 |
| Ihmiset liikkuu paljon eli en ihmettele että pandemia tuli |  | 1 | 2 |
| Yhteiskunnalliset asiat huolestuttaa |  | 1 | 1 |
| SYYTTELYÄ |  | 0 | 0 |
| Korona on ihmisten kulutuksen syytä |  | 1 | 1 |
| Kova ryhmäpaine ja syylistämistä |  | 0 | 0 |
| Kova ryhmäpaine jälkikaranteniin ja muuhunkin ohjeistukseen |  | 1 | 2 |
| Leima on varmasti kun kävelin ulkona naapuri antoi ymmärtää että ei pitäisi |  | 1 | 1 |
| Poliiseina ne nuoret jotka ei mene ulos N |  | 1 | 1 |
| Poliiseina olevat eivät ajattelekokonaiskuvaa että ihmisillä on eri elämäntilanteita ja eri sos tarpeita kuten yksinasujat |  | 1 | 1 |
| Poliisina olevat ajattelee että on epäreilua jos joku ei noudata |  | 1 | 1 |
| Sisko kysyi miten kertaat mennä kauppaan karanteenin jälkeen |  | 1 | 2 |
| Sisko oli aika poliisina varsinkin alkuaikoina että en sais mennä kauppaan tai nöin |  | 1 | 2 |
| Sosiaalinen paine on kova eli haluttais lähteä ulosa ja tavata kavreita mutta pelottaa mitä jotku sanoo tai ajattelee |  | 1 | 1 |
| Syylistämistä Whats App ryhmissä joissa ei itse ole mukana mm oman asuinalueen |  | 1 | 1 |
| Syyttelyä etäisiltä tutuilta että eihän konserttia olisi pitänyt pitää |  | 1 | 1 |
| Syyttelyä koulun vanhempien keskuudessa joita emme välttämättä tunne |  | 1 | 1 |
| Syyttelyä media jutun jälkeen että miksi järjetsää häitä korona aikaan tai miksi mennä häihin koronan aikaan tai miksi tulla ulkomailta tartuttamaan häävieraita |  | 1 | 1 |
| Ulkona mulkoillaan tosia että mitäs säkin olet täällä kävelemässä |  | 1 | 1 |
| Ulkopiiri syytteli vain meitä |  | 1 | 1 |
| yllättävän vähän syyttelyä mahdollisesta tartuutamisesta työyhteisössä |  | 1 | 3 |
| TEINIT |  | 0 | 0 |
| epämiellyttävä olo kun vaotiinn niin kuin meillä olis rutto |  | 1 | 1 |
| Huolta vasta siinä vaiheessa kun kuuli tutun vakavista oireista |  | 1 | 1 |
| Puhutaan paljon ja pohditaan kolua |  | 1 | 3 |
| Puhutaan sillä ajatuksella että korona ei ole vakavaa |  | 1 | 1 |
| UTELIAISUUTTA |  | 0 | 0 |
| Aikuisia kiinnostaa tosi paljon oireet |  | 1 | 1 |
| Ihmiset on uteliata ja haluaa tietää koronasta |  | 1 | 1 |
| Ihmiset on tosi kiinnostuneita |  | 1 | 3 |
| Ihmisillä on tarve omakohtaiseen tietoon |  | 1 | 1 |
| Sairaalassa tuli kymmenen hoitajaa kysymyään miltä tuntuu |  | 1 | 2 |
| Työkaverit erityisen kiinnostuneita oireista |  | 1 | 1 |
| Uteliaita viiiestejä myös ihmisiltä joita emme oikeastaan edes tunne |  | 1 | 1 |
