# Supplementary material for: Learning about COVID-19-related stigma, quarantine and isolation experiences in Finland
Source: PLoS One. 2021 Apr 14;16(4):e0247962. doi: 10.1371/journal.pone.0247962 (PMC8046198; doi:10.1371/journal.pone.0247962)
Supplement: S1 Question guide — (DOCX) [file pone.0247962.s002.docx]

**Discussion with coronavirus quarantined or isolated family members**

1. **Let us start talking about your experiences with quarantine / isolation?**
   1. **Probes: How did it all started? What happened next?**
2. **How did people treat you during your quarantine/ isolation?**
   1. **Probes: Can you tell me some examples of situations in which you felt being treated negatively?**
3. **How do you think these experiences impacted you?**
   1. **Can you give me some concrete examples**
4. **What do you think people in generally think of people associated with coronavirus in Finland?**
5. **What did you think about yourself during quarantine / isolation?**
   1. **Probes: What kind of negative feelings and positive did you have towards self ?**
6. **From where do you think these feelings came from?**
7. **How did these negative feelings impact you ?**
8. **What can you tell me about your everyday life and routines during quarantine/ isolation ?**

**Probe: Tel me about your daily routines? How did they differ from you daily routines before quarantine/isolation? Did you make special arrangements to avoid contacts with other household members?**

1. **From where and how did you receive COVId19 and quarantine/ isolation related information?**
2. **What can you tell me about your everyday life and routines during quarantine/ isolation?**

**Probe: you make special arrangements to avoid contacts with other household members?**

1. **Tell me about COVID-19 and quarantine/ isolation related information that you received during quarantine/isolation.**
2. **What has been the most memorable or significant issue in your quarantine/isolation experience?**
